# Hope and the Life Course: Results From a Longitudinal Study of 25,000 Adults

**DOI:** 10.1002/hec.70041

**Published:** 2025-10-07

**Authors:** Carol Graham, Redzo Mujcic

**Affiliations:** ^1^ Brookings Institution Washington District of Columbia USA; ^2^ University of Maryland College Park Maryland USA; ^3^ Warwick Business School University of Warwick Coventry UK

**Keywords:** adaptation, hope, life course, longitudinal analysis, wellbeing

## Abstract

This paper reports the first large‐scale longitudinal links between one of the least known dimensions of wellbeing—hope—and long‐term outcomes in a range of life arenas. Hope has agentic properties which are relevant to people's future outcomes. Following 25,000 randomly sampled Australian adults over a period of 14 years from 2007 to 2021 (*N* > 115,000), we find a strong link between hope and better contemporary and future outcomes. Individuals with high levels of hope had improved later wellbeing, education, economic and employment outcomes, perceived and objective health, and are less likely to be lonely. Hope is associated with higher resilience, ability to adapt, and internal locus of control. It also serves as a psychological buffer during bad times. Respondents with high levels of hope were less likely to be influenced by negative life events and adapted more quickly and completely after these major events. Better understanding the drivers and consequences of hope can ultimately inform public policy to improve people's lives.

*Hope is not a Promise We Give; it is a Promise We Live*

Amanda Gorman, 2021

*Hope is a Waking Dream*

Aristotle

## 1 Introduction

Emotions are central to the study of human well‐being. Yet mainstream economists have only begun to think about the importance of these factors in economic, social, and health behaviors. Hope is likely the most important positive emotion and socio‐emotional trait directly relevant to long‐term outcomes (Graham 2023; Edwards et al. 2025) but is the least studied dimension of well‐being. It is distinguished by its strong grounding in individual agency. Hope is not just a belief that things will get better (i.e., optimism), but the determination to make them better, which reflects agency and determination (grit); see Snyder (1995) and Graham (2023). The distinction between tragic optimists and hopeful pessimists (Brooks 2021) is another way to think of this.

As a result, of all the well‐being dimensions, hope seems to be the most critical to future‐oriented behaviors and to long‐term outcomes. Hope, like happiness and other aspects of well‐being, has a genetic component and is also shaped by environmental factors such as familial and community support, education, and opportunity (DeNeve et al. 2012). Socio‐emotional traits—like hope—and skills, unlike cognitive skills which are not malleable after the late twenties and early thirties, can be developed throughout the life‐course (Heckman and Kautz 2012).

There is a fledging literature in economics and other social sciences that focuses on hope (Graham 2023; Graham and Ruiz Pozuelo 2023; Long et al. 2024; O'Connor and Graham 2019; Pleeging et al. 2021; Rozanski et al. 2019; Dursun and Cesur 2016; for a summary of the literature in psychiatry, see Schrank et al. 2008). Much of that literature finds empirical evidence of the linkages between hope, future oriented behaviors, and better long‐term outcomes, in the economic, education, health, and social arenas. Snyder's (1995) well‐known definition of hope, which is the belief that one can make things better, highlights the role of individual agency and having a path forward to better future outcomes as integral to the concept; education, as above, is an important pathway. Psychiatrists often cite restoring hope as the critical first step to recovery from mental illness, but do not provide details on how it operates, and whether hope acts as a moderator when it comes to well‐being outcomes (which we explore statistically in this paper).

Recent research has tracked lack of hope as a key factor in the rising numbers of U.S. deaths of despair—a term that encompasses suicide, overdoses, and alcohol‐related mortality (Graham and Pinto 2019). Surveys of hope among low‐income adolescents in Lima, Peru, and St. Louis, Missouri, have shown that those who have hope for the future—and who are often supported by a mentor—are more likely to invest effort in their education and avoid risky behaviors (Graham and Ruiz Pozuelo 2023). By contrast, individuals in despair (who, by definition, have lost all hope) are unlikely to respond to incentives or opportunities and are vulnerable to misinformation and conspiracy theories as well as to despair‐related deaths (Graham 2024).

The present paper seeks to contribute to and deepen the extant knowledge in that literature by using a survey question that measures hope in a large‐scale longitudinal data set from Australia—the HILDA Survey panel. The paper primarily explores two main research questions.

1. Does hope act as a psychological buffer against major life events over the life course?
2. Do higher levels of hope lead to improved future outcomes over the life course?

To shed light on these empirical questions, there are two key scientific requirements or obstacles in place. First, we need to be able to survey and follow the same set of individuals over long periods of the life course. Second, we need access to quality micro‐level data and information about major life events and shocks experienced by the sampled individuals. To our knowledge, currently there exists no such large‐scale longitudinal analysis on human feelings of hope. The present study overcomes the above methodological demands by following a random sample of approximately 25,000 Australian adults through time—over a period of 14 years—and thus provides some of the first scientific evidence of its kind.

## 2 Data and Methods

### 2.1 Data

For our data, we rely on the Household, Income and Labor Dynamics in Australia (HILDA) Survey, which is a large scale, nationally representative data set from Australia. The longitudinal HILDA panel collects annual information from members of Australian households who are at least 15 years of age (see Wooden and Watson 2007). Data are collected each year by face‐to‐face interviews and self‐completion questionnaires. The former technique is mainly used to record demographic and socio‐economic information, while the latter approach is used to measure respondent attitudes and wellbeing, workplace and health outcomes, and lifestyle choices. Overall, quality individual‐level information is collected on a variety of general and specialized topics including labor market dynamics, income, education, family composition, as well as the physical and psychological well‐being of individuals.

The HILDA Survey began providing information on a total of 13,969 individuals from 7682 different households interviewed since the first survey wave in 2001. A large top‐up sample of 2153 new households and 4009 individuals occurred in 2011 (wave 11). This was done to improve representativeness and to correct for changes in household composition.

The present analysis draws upon eight waves of the HILDA over 14 years, beginning in 2007 and then repeated in 2009, 2011, 2013, 2015, 2017, 2019, and 2021. Across these survey years, the total number of respondents is 36,252.

It is also in these survey waves that the unique measure of feelings of hope is available. After excluding respondents with missing information on the key outcome variables and other covariates, the available sample is approximately 25,700 respondents, aged 15 to 101, and 115,600 person‐year observations.

For this final sample of individuals, the average duration in the panel is 4.5 survey waves. About 20% (*n* = 5372) of the respondents are present during the entire observation period, that is, in each of the eight waves from 2007 to 2021. Slightly more than 40% (*n* = 10,785) of respondents are present in at least six of the survey waves. And 80% (*n* = 20,577) of the respondents are recorded for two waves or longer. We later use this longitudinal information to test how people's feelings of hope are linked to future outcomes, both in the near and more distant periods (i.e., 2, 4, and 10 years ahead).

Sample attrition in the HILDA panel is extremely low, when compared to other household panel surveys from around the world. The annual reinterview rates of sample members are more than 94% since the fifth wave in year 2005 (see Watson et al. 2018).

Our measure of hope is based on the reverse coding of a question on psychological distress in the survey: “*In the past 4 weeks, how often have you felt hopeless*?”, with possible answers ranging from “All of the time”; “most of the time”; “some of the time”; “a little of the time”; and “none of the time”. The distribution of responses to this question and hope variable, which is asked biannually in waves 2007 to 2021, is reported in Table 1 below. The median response to this question is equal to 5, and the distribution of responses is negatively skewed, with approximately 80% of the responses scored as 4 and above. Overall, the average self‐reported level of hope is 4.52 with a standard deviation of 0.84 (see Table 2).

**TABLE 1:** Distribution of hope measure, HILDA survey 2007–2021.

Felt hopeful (in the past 4 weeks)	Frequency	Percent	Cumulative frequency
1. None of the time	1013	0.88	0.88
2. A little of the time	3248	2.81	3.68
3. Some of the time	10,083	8.72	12.40
4. Most of the time	21,142	18.28	30.69
5. All of the time	80,145	69.31	100.00
Total	115,631	100.00	

**TABLE 2:** Summary of hope and well‐being measures, HILDA survey 2007–2021.

	Mean	SD	Min	Max
Felt hopeful	4.52	0.84	1	5
Life satisfaction	7.94	1.42	0	10
Been a happy person	4.37	1.10	1	6
Locus of control	37.88	8.01	7	49

*Note:* The distribution of the hope scores is reported in Table 1 (*N* = 115,631). Overall life satisfaction scores are based on the question: *All things considered, how satisfied are you with your life?* (*N* = 115,585). *Been a happy person* is a psychological distress measure on how often the respondent felt happy over the past 4 weeks (*N* = 114,895). Responses related to the measures of hope, life satisfaction, and happiness are available in the HILDA Survey for years 2007, 2009, 2011, 2013, 2015, 2017, 2019, and 2021. Locus‐of‐control questions are only available in the HILDA Survey for years 2007, 2011, 2015, and 2019 (*N* = 56,760).

While there is less accumulated experience with hope survey questions (and with different phrasing and scales), our analysis—and those of the previous studies cited above—suggest that regardless of the specific question framing and scales, the existing hope questions all track in a similar fashion and area also validated by psychometric measures such as Duchenne smiles and frontal lobe activity. Higher scores on many of these questions are associated with a variety of better long‐term outcomes (Diener 1984). Hope is also not simply an analog of anxiety or depression, as it displays agentic properties that the affective questions do not yield.

Closely relevant to the approach used in the present study is the recent longitudinal work, also based on the HILDA survey, by Kaiser and Oswald (2022)—demonstrating a clear statistical link between reported feelings and later chosen actions by the same respondents.

Other wellbeing variables, that we also include in Table 2 and later analysis, are “Overall life satisfaction” based on the usual 0–10 scale, and “Been a happy person” (on a 1–6 scale). While life satisfaction is an evaluative measure, happiness can be an evaluative or an affective measure, depending on the time frame that is posed. Our primary focus is on the life satisfaction question, but we also used the “Been a happy person” variable as a supplemental question, as the longer time frame of the question makes it closer to an evaluative than affective measure.

Demographic variables are age, gender, marital status, number of children, number of adults in the household, home ownership, resides in major city, regional, or rural area, Aboriginal or Torres Strait Islander origin, and migrant. These are summarized in Supporting Information S1: Table A1.

Education level is determined by university degree(s), diploma, certificate, high school graduate, and high school dropout. Employment indicator variables are unemployed, employed full‐time, employed part‐time, not in the labor force, and retired. Income variables are hours per week in main job, household disposable income, and neighborhood socio‐economic status as captured by IRSAD (an Australian census‐based Index of Relative Socio‐Economic Advantage and Disadvantage, which assesses the economic and social conditions in a particular geographical area). The latter neighborhood index is primarily derived by aggregating household income and occupation levels within a local government area.

As reported in Supporting Information S1: Table A2, health variables include long‐term health condition or disability, self‐assessed health, body mass index (BMI), alcohol drinker/heavy drinker, smoker/heavy smoker, and number of hospital admissions in the past year. Social life conditions are social person, number of visitors, need help but do not get it, number of friends, no one to confide in, no one to lean on, someone to cheer me up, and feel very lonely.

An additional variable covers the likelihood of taking financial risk. The question is “which of the following statements comes closest to describing the amount of financial risk that you are willing to take with your spare cash? i.e., cash used for savings or investment” with possible answers ranging from “[1] not willing to take financial risks” up to “[4] takes substantial risk expecting substantial returns”. The average score for the financial risk‐taking measure is 1.63 with a standard deviation of 0.70.

Finally, we look at positive and negative life events to compare differences in the ability to adapt between those individuals with high hope and those with low hope. The questions about these events in the HILDA data include major disturbances to personal finances (e.g., winning the lottery, receiving an inheritance, or going bankrupt), changes (positive or negative) on the employment front, pregnancy and the birth of a child, marital changes such as divorce or separation, the death of a spouse, child, or friend, injuries and serious illnesses, victim of a crime, incarceration (of the respondent or of a family member), and weather‐related disasters such as the loss of a home.

Specifically, respondents are told:

We now would like you to think about major events that have happened in your life over the past 12 months. For each statement cross the YES box or the NO box to indicate whether each event happened during the past 12 months. If you answer “YES”, then also cross one box to indicate how long ago the event happened or started. This information is given by quarter.

These life events are summarized in Table 3. In the full sample, we observe around 130,000 reported life events, with the most common event being “moved house” (17% of the sample) followed by “a family member being physically harmed” (14%), the “death of a friend or relative” (11% for each life event), and “serious personal injury or illness” (9%). The death of a spouse or child is the least common event (1%), closely followed by “losing a home to a natural disaster” (2%). A similar low frequency is observed for major financial gains and losses, with each making up about 3% of all reported life events. The “change of job” is a more common life event (13%) and can be either positive or negative, depending on the circumstances.

**TABLE 3:** Summary of major life events, HILDA survey 2007–2021.

	Mean	SD	Min	Max
Money gained (e.g., won lottery)	0.03	0.17	0	1
Money lost (e.g., went bankrupt)	0.03	0.16	0	1
Hired (changed jobs)	0.13	0.34	0	1
Fired	0.03	0.18	0	1
Promoted	0.06	0.24	0	1
Retired	0.02	0.16	0	1
Married	0.02	0.14	0	1
Pregnant	0.06	0.23	0	1
Childbirth	0.04	0.19	0	1
Separated	0.04	0.19	0	1
Reconciled	0.01	0.10	0	1
Moved house	0.17	0.37	0	1
Spouse/child died	0.01	0.09	0	1
Friend died	0.11	0.31	0	1
Relative died	0.12	0.32	0	1
Health shock	0.09	0.28	0	1
Attacked	0.01	0.12	0	1
Family harmed	0.14	0.35	0	1
Jailed	0.00	0.05	0	1
Relative jailed	0.02	0.13	0	1
Home lost to natural disaster	0.02	0.13	0	1

*Note:* Individual‐level data on major life events are also available in the HILDA Survey for years 2007, 2009, 2011, 2013, 2015, 2017, 2019, and 2021 (*N* = 115,630). Respondents are specifically asked: *We now would like you to think about major events that have happened in your life over the past 12 months. For each statement cross the YES box or the NO box to indicate whether each event happened during the past 12 months. If you answer “YES,” then also cross one box to indicate how long ago the event happened or started. This information is given by quarter.*

### 2.2 Methods

We rely on the standard econometric techniques that are commonly used in the longitudinal analysis of subjective wellbeing data. The specific regression equations that we estimate and analyze are described in the corresponding results sections and output tables below. The main dependent variables are changes in hope and life satisfaction, and long‐term outcomes associated with current levels of hope.

In general, we primarily use fixed‐effects (within‐person) panel regressions—an approach that allows us to eliminate time‐invariant unobserved individual factors and helps ensure that any observed relationship is not just a spurious cross‐sectional pattern caused by omitted confounding factors such as personality, family upbringing, or background wealth.

We also treat the data as if from a prospective setting. Here the regression equations reveal that a higher level of hope in the current period is associated with better life outcomes—measured across various economic, social, and wellbeing domains—in the future even after controlling for current or starting levels of those life outcomes.

## 3 Results

### 3.1 Hope Dynamics and Major Life Events

One major question in our analysis is the extent to which hope serves as a psychological buffer and helps people adapt more quickly to negative life events.

Before looking into this question and analysis more closely, we first estimate the average effect of major life events on self‐reported hope levels to get some idea about the dynamics of hope—and specifically how feelings of hope evolve before, during, and after such life events. In general, for the latter, our findings run in the same direction as those in earlier studies focusing on human happiness and life satisfaction, in which negative events typically take longer to adapt back from then positive events (Clark et al. 2008; Frijters et al. 2011; Oswald and Powdthavee 2008; Etilé et al. 2021; Johnston and Stavrunova 2021).

Life events that have the most negative momentary effect on hope are major financial losses, being seriously ill or injured, the death of a spouse or child, experiencing a serious physical injury or being a victim of physical violence, and being incarcerated (see Supporting Information S1: Tables A4 and A7 in the online appendix). Rather surprisingly, while the onset of a pregnancy has positive effects on hope, the effect seems to fade with the birth of a child. For a detailed supplementary analysis of other socioeconomic determinants of hope, see the summary and Supporting Information S1: Tables A3‐A6 in the online appendix.

We also explore the effects of leads (anticipation of) or lags on hope to explore differences in adaptation, looking at patterns in hope both 2–4 years before and 2–4 years after life events (Supporting Information S1: Figure A3; Table A7). We find that the average respondent adapts to almost all life events by 2 years after the events. For major financial losses, e.g., the most negative changes in hope are in the year leading up to the loss (i.e., the momentary effect) and fade away about 2 years after the event. A similar adaptation period is apparent for marital separation or divorce. There is also clear evidence that individuals actively anticipate bad events, such as major financial losses, in the 2–4 years beforehand (see Supporting Information S1: Figure A3.B). For life events related to individual health, such as serious personal injury or illness, adaptation in feelings of hope does not fully take place even 4 years later.

A clear gender difference apparent in Supporting Information S1: Figure A4 is that females tend to anticipate being a “victim of physical violence” well before it takes place. These strong lead effects and reductions in hope may be explained by such events being cases of domestic violence by the same partner or perpetrator.

Significant job and income‐related changes have more negative effects on feelings of hope for the poor than for the rich (see Supporting Information S1: Figure A5), likely because the poor have far less of a financial buffer to weather such events. There are also some modest differences between the poor and the rich on other major life events, such as marriage, the death of a child, being a victim of physical violence, losing a home to natural disaster, or incarceration (see Supporting Information S1: Figure A5; Table A9). In all these cases, except for marriage which has a more positive effect on hope for the poor than the rich, these events unsurprisingly also have a more negative effect, likely because the poor have less resources to serve as a buffer in the face of events which harm their health or family situation (see also the recent study from Indonesia by Escobar Carias et al. 2022).

### 3.2 Hope as a Psychological Buffer

We then compared the adaptation times of those individuals with high and low levels of hope. Our main outcome variable of interest here is overall life satisfaction—as summarized in Table 2. For this exercise, we re‐scaled the hope variable into those with the highest levels of hope (subtracting 1 from the 1–5 scale on the variable and dividing by 4). With the resulting category 0 being very low hope and 1 being the highest hope. Roughly 1% of the sample were in the lowest category and 69% were in the highest.

Using this variable, a one‐unit change reflects the difference from being in the lowest hope category to the highest (or totally hopeless to totally hopeful). In Table 4, we first explored differences in the effects of negative life events on those with the highest levels of hope (standardized hope score equal to 1 on a 0–1 scale) compared to the rest of the sample, in separate fixed effects regression with those with low (standardized hope score ≤ 0.25) and high levels of hope (standardized hope score > 0.5) and using life satisfaction as the dependent variable. Overall, we estimate three separate fixed‐effects life satisfaction equations—one for each different category or strength of hope (see Table 4, columns 1–3). The estimated life satisfaction equations control for the standard set of time‐varying covariates including age, income, education, marital status, and survey‐year dummies.

**TABLE 4:** Effect of negative life events on life satisfaction by hope level, HILDA survey 2007–2021.

Dependent variable:	(1)	(2)	(3)	Test of difference:
Life satisfaction	Low hope	High hope	Full of hope	(1) versus. (3)
Money lost	−0.553**	−0.388**	−0.297**	**
	(0.177)	(0.035)	(0.042)	
Fired	−0.056	−0.045	−0.028	**
	(0.202)	(0.024)	(0.026)	
Separated	−0.162	−0.163**	−0.123**	**
	(0.203)	(0.027)	(0.032)	
Spouse or child died	−0.391	−0.207**	−0.171*	*
	(0.378)	(0.063)	(0.068)	
Illness	−0.273	−0.173**	−0.153**	**
	(0.143)	(0.016)	(0.017)	
Physically attacked	−0.235	−0.118	−0.063	**
	(0.230)	(0.049)	(0.059)	
Jailed	−0.192	−0.157	−0.051	
	(0.602)	(0.143)	(0.183)	
Lost home to disaster	−0.178	0.053	0.027	
	(0.273)	(0.030)	(0.032)	
No. of observations	3811	89,497	70,672	74,483
No. of individuals	2592	22,942	20,145	21,515

*Note:* Figures are coefficient estimates from three separate fixed‐effects (within‐person) models of overall life satisfaction by level of hope. These regressions should be read vertically. “Low hope” subsample includes respondents with a standardized level of hope ≤ 0.25 (on a 0–1 scale). “High hope” subsample includes respondents with a standardized level of hope > 0.5. “Full of hope” subsample includes respondents with a standardized level of hope that equals 1. Standard errors clustered at the individual level are presented in parentheses. Life satisfaction measure ranges from 0 (totally dissatisfied) to 10 (totally satisfied) and is summarized in Table 2. Life events are summarized in Table 3. Included in the life satisfaction equations but not shown are the covariates age, education, marital status, household composition, employment status, household income, neighborhood SES index, homeownership status, residential area, and year dummies. The average age in the full sample is 45.4 years (ranging from 15 to 101). Age distribution of respondents: 17% (15–24); 17% (25–34); 16% (35–44); 17% (45–54); 15% (55–64); 11% (65–74); 7% (≥ 75 years old). *p*‐values are corrected using the Bonferroni method (see Bland and Altman 1995). In this table, we multiply the original *p*‐values by 3; as we have 3 separate regression‐equations (columns)—across which we compare the estimated life‐event coefficients. Two‐sided *test of difference* is based on interaction terms from a fixed‐effects life satisfaction equation—using the subsample of low‐hope and full‐of‐hope individuals only. The estimated regression includes interaction terms between the negative life events and a binary indicator for whether or not the individual is in the low‐hope group. * and ** denote statistical significance at the 5% and 1% levels, respectively.

We find that those respondents with the most hope experience the weakest negative effects on life satisfaction from negative life events, and those with the lowest levels the greatest (almost twice as large as the most hope group), with the general hope group in the middle. For example, the slope coefficient on the “money lost” indicator variable—that captures negative financial events such as bankruptcy—is estimated to be −0.553 for the “low hope” subsample and −0.297 for the “full of hope” subsample (see Table 4). This difference is statistically significant at the 1% level. Thus, respondents with a lot of hope appear to undergo much smaller falls in their life satisfaction levels compared to those respondents with not much hope at all. A similar psychological buffer is apparent for high‐hope individuals given many of the other life events estimated in Table 4 including job loss; divorce; death of a spouse or child; becoming seriously ill; and being physically attacked.

**FIGURE 1:**
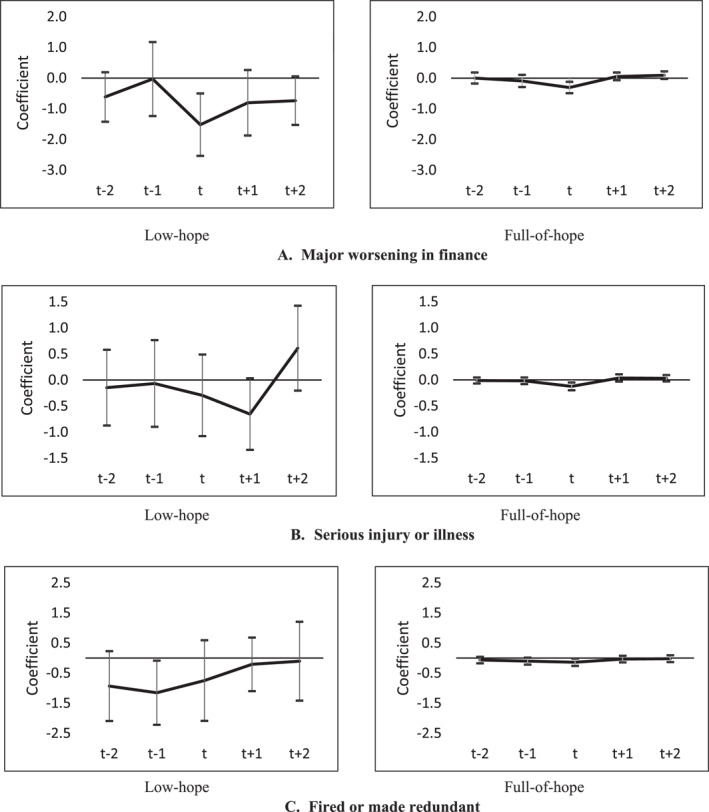
Leads and Lags in Life Satisfaction to Negative Life Events by Hope Level, HILDA 2007–2021. Each value on the vertical *y*‐axis represents the lead and lag coefficients of the major life events as reported in Supporting Information S1: Table A10. “Low hope” subsample includes respondents with a standardized level of hope ≤ 0.25 (on a 0–1 scale). “Full of hope” subsample includes respondents with a standardized level of hope that equals 1. The life event of interest took place at year *t = 0*. Periods *t − 1* and *t − 2* capture 2–3 years and 4–5 years before or leading up to the event, respectively. Periods *t + 1* and *t + 2* capture 2–3 years and 4–5 years after or following the event, respectively. All reported life events are summarized in Table 3. Life satisfaction measure ranges from 0 (totally dissatisfied) to 10 (totally satisfied) and is summarized in Table 2. Vertical lines represent 95% confidence intervals at each time period.

In Figure 1, we also analyzed differences in adaptation times. The estimated profiles and hope dynamics are much flatter, and closer to the zero line, for the full‐of‐hope subsample. The latter group recovered more quickly from negative life events than the low‐hope group, particularly after major worsening in finances. From Figure 1, there is also evidence of strong anticipation effects when it comes to some life events—such as being fired or made redundant from a job—again indicating that our measure of hope is picking up people's actual feelings quite some time before the event takes place.

### 3.3 Hope and Long‐Term Outcomes

The most fundamental question in our analysis is: does having hope today result in better outcomes in the future? Our results suggest that the answer to this is a strong yes. While other recent studies (e.g., Graham and Ruiz Pozuelo 2023; Long et al. 2024; O'Connor and Graham 2019) have found this to be the case, the HILDA survey allows for a much deeper exploration of the life course based on an unusually large longitudinal data set covering 14 years.

We estimate regressions based on a framework which looks at having hope today on a range of individual outcomes in future years, controlling for the same covariates as in the earlier regressions and time frames running from *t* + 1 (2 years), *t* + 2 (4 years), and *t* + 5 (10 years) periods later, based on the available waves of the HILDA panel. We use a prospective analysis approach which looks at hope today and different outcomes in subsequent years, controlling for the starting level of the variable of interest in the current year. Since accounting for the latter value on the right‐hand side of the regression equation is crucial, and standard fixed‐effects specifications are biased (Nickell 1981), we use a simple pooled OLS approach. This kind of prospective analysis is widely used across the social sciences (e.g., Bridger and Daly 2020; Long et al. 2024; Mujcic and Oswald 2018).

Our results in Table 5 show that having hope today is associated with higher levels of well‐being (both life satisfaction and “been a happy person”) in future years, controlling for initial levels of these same variables.

**TABLE 5:** Estimated effect of current hope on future life outcomes, HILDA survey 2007–2021.

	Years into the future:		
Outcome variables:	+2 years	+4 years	+10 years
Wellbeing:			
Life satisfaction	0.628**	0.612**	0.683**
	(0.030)	(0.038)	(0.064)
	*N = 85,027*	*N = 67,109*	*N = 25,579*
Been a happy person	0.652**	0.577**	0.540**
	(0.024)	(0.029)	(0.051)
	*N = 84,024*	*N = 66,346*	*N = 25,286*
Education:			
University degree (bachelor's)	0.042**	0.043**	0.044
	(0.010)	(0.011)	(0.015)
	*N = 66,990*	*N = 53,752*	*N = 21,622*
Economic:			
Unemployed	−0.021**	−0.020**	−0.016
	(0.004)	(0.005)	(0.007)
	*N = 69,990*	*N = 53,752*	*N = 21,622*
Earnings (disposable income)	6529.8**	7414.7**	16,607.2**
	(898.6)	(1245.4)	(2403.2)
	*N = 67,195*	*N = 53,925*	*N = 21,712*
Neighborhood SES	0.060	0.116	0.186
	(0.026)	(0.039)	(0.085)
	*N = 85,308*	*N = 67,337*	*N = 25,697*
Changed jobs	−0.028**	−0.028*	−0.015
	(0.007)	(0.008)	(0.012)
	*N = 66,378*	*N = 53,287*	*N = 21,442*
Financial risk taking	0.033	0.046	−0.013
	(0.016)	(0.021)	(0.050)
	*N = 49,793*	*N = 37,130*	*N = 7402*
Health:			
Poor health	−0.138**	−0.142**	−0.158**
	(0.007)	(0.009)	(0.016)
	*N = 83,388*	*N = 65,820*	*N = 25,025*
BMI: Obese	−0.033**	−0.025	−0.045
	(0.006)	(0.008)	(0.016)
	*N = 85,084*	*N = 67,151*	*N = 25,601*
Heavy drinker	0.003	0.011	0.014
	(0.004)	(0.006)	(0.011)
	*N = 85,084*	*N = 67,151*	*N = 25,601*
Heavy smoker	−0.032**	−0.035**	−0.023
	(0.005)	(0.006)	(0.012)
	*N = 85,084*	*N = 67,151*	*N = 25,601*
Serious injury or illness	−0.061**	−0.064**	−0.059**
	(0.006)	(0.007)	(0.012)
	*N = 83,827*	*N = 66,230*	*N = 25,277*
Social:			
Felt very lonely	−1.279**	−1.243**	−1.159**
	(0.038)	(0.045)	(0.078)
	*N = 83,719*	*N = 66,120*	*N = 25,202*
Have lots of friends	0.404**	0.410**	0.420**
	(0.029)	(0.035)	(0.064)
	*N = 83,777*	*N = 66,177*	*N = 25,219*
Jailed	−0.004**	−0.004	−0.006
	(0.001)	(0.001)	(0.003)
	*N = 84,066*	*N = 66,405*	*N = 25,339*

*Note:* Prospective analysis of future life outcomes as a function of current feelings of hope. These regressions should be read horizontally. For each listed dependent (outcome) variable, we estimate three separate pooled OLS models predicting the outcome variable *t + 2*, *t + 4*, and *t + 10* years into the future. Robust standard errors clustered at the individual level are presented in parentheses. The average age in the full sample is 45.4 years (ranging from 15 to 101). Age distribution of respondents: 17% (15–24); 17% (25–34); 16% (35–44); 17% (45–54); 15% (55–64); 11% (65–74); 7% (≥ 75 years old). For the economic and educational outcomes (except Neighborhood SES), we restrict the sample to individuals aged between 18 and 65. Included and controlled for in each prospective model but not shown is the level of the outcome variable in the current year *t* as well as the covariates age, education, marital status, household composition, employment status, household income, long‐term health condition, neighborhood SES index, homeownership status, residential area, and year dummies. As an example, for the future outcome of graduating with a bachelor's degree—the estimated model controls for and is conditional on the individual having graduated from high school in the current year. *p*‐values are corrected using the Bonferroni method (see Bland and Altman 1995). In this table, we multiply the original *p*‐values by 48; as we have 16 separate regression‐equations (outcomes) over 3 time periods. * and ** denote statistical significance at the 5% and 1% levels, respectively.

We next explore the effects of hope today on education, economic, health, and social outcomes, with the same specification. We find that “moving” from totally hopeless to totally hopeful results in a 4‐percentage‐point higher probability of achieving a bachelor's degree in the next 2 years, and also a 4‐percentage‐point boost in the probability of achieving one in the next 4 years. We also find that the effect of hope on future investment into education is more important for the poor than the rich (see Supporting Information S1: Tables A13 and A14), which is intuitive given that the poor are less likely to have the means to achieve higher education than the rich and need to work harder to do so (Graham and Ruiz Pozuelo 2023 also find this to be the case in Peru).

In the economic realm, moving from totally hopeless to totally hopeful reduces the probability of being unemployed in future years by around 2 percentage points, and increases the likelihood of having higher earnings and living in a higher SES neighborhood in the future. The latter finding—on moving to a richer part of town—statistically only holds for individuals from poor households, 10 years from today (see Supporting Information S1: Table A13). Higher hope is linked to a lower probability of switching jobs in future years, but not to being more likely to take financial risks. We note that, based on intuition, much of the economic and work‐related analysis is restricted to respondents aged between 18 and 65; except for living in a more prosperous neighborhood, which can generally occur at any age.

In the health realm, higher levels of hope are linked to higher levels of self‐reported health, a lower probability of being obese (in the next 2 years; but, as expected, this result decreases in size and becomes statistically insignificant after we control for self‐assessed health in Supporting Information S1: Table A17), to reductions in smoking levels, and to a lower likelihood of having a serious illness or injury. The only health arena where hope seems to have no significant effect is on levels of alcohol consumption. In the social arena, higher levels of hope are linked to a lower probability of being lonely, a higher one of having many friends, and a somewhat lower probability of being incarcerated in future years (see Table 5 for statistical details).

We still do not fully understand the underlying channels or actual mechanisms by which hope affects long‐term outcomes and actions. Yet our analysis is suggestive of hope's role as a psychological buffer (i.e., a moderator), its associated qualities of agency and resilience, and the incentives that beliefs in better futures provide for investments in those futures and for avoiding risks that jeopardize them. One possible channel that we describe in greater detail in the next section is internal locus of control.

### 3.4 Hope and Internal Locus of Control

In this section, we explore the role of internal locus of control as an important channel that connects and drives the relationship between hope and long‐term outcomes, in keeping with Snyder's (1995) definition as hope being comprised of aspirations, pathways, and agency. We posit that those with hope also have higher levels of internal locus of control, which helps them recover from negative life events and continue to invest in their futures, among other things. In our earlier surveys in Peru, we found that hopeful adolescents had higher levels of locus on control that those with low hope.

The HILDA survey fielded a locus of control question in survey years 2007, 2011, 2015, and 2019. The questions that are used to measure it are: “I have little control over the things that happen to me; There is really no way I can solve some of the problems I have; There is little I can do to change many of the important things in my life; I often feel helpless in dealing with the problems of life; Sometimes I feel that I'm being pushed around in life; What happens to me in the future mostly depends on me; I can do just about anything I really set my mind to do.”

Following Buddelmeyer and Powdthavee (2016), we compute the locus‐of‐control score by adding the responses to questions 1 through 5, subtracting the scores from questions 6 and 7, and adding a constant of 16. Using this metric, the locus‐of‐control (LOC) variable ranges between 7 (internal) and 49 (external). For ease of interpretation, we reverse these scores so that a higher value represents relatively more internal locus of control. The mean score for this LOC measure is 18.1, with 49 being the highest. We standardized the variable (LOC) as a 0–1 variable, with those above the mean having higher than average internal locus of control. Supporting Information S1: Figure A2 plots the distribution of the standardized locus of control variable.

We first ran our standard hope regression (both pooled cross section and fixed effects) with LOC as a covariate and it has positive, significant, and large coefficients in both specifications (see Supporting Information S1: Table A11). We next ran separate fixed‐effects hope regressions in Table 6 for those in the low LOC category and those in the high one, with low LOC being the bottom 25% on the LOC distribution and high LOC being the top 25%. Those individuals with high LOC suffer less lost hope from most negative life events and recover more quickly from them. The latter is found to be especially true for life events such as “serious personal injury or illness” and “victim of physical violence” (see Supporting Information S1: Figure A6).

**TABLE 6:** Effect of negative life events on hope by locus of control type, HILDA survey.

Dependent variable:	(1)	(2)	(3)	Test of difference:
Felt hopeful	Full sample	External LOC	Internal LOC	(2) versus. (3)
Money lost	−0.292**	−0.302**	−0.107	**
	(0.020)	(0.071)	(0.062)	
Fired	−0.047**	−0.115	−0.009	**
	(0.016)	(0.089)	(0.036)	
Separated	−0.134**	−0.103	−0.060	**
	(0.018)	(0.090)	(0.044)	
Spouse or child died	−0.161**	−0.375*	−0.024	**
	(0.033)	(0.147)	(0.042)	
Illness	−0.120**	−0.230**	−0.028	**
	(0.010)	(0.042)	(0.021)	
Physically attacked	−0.206**	−0.300*	0.066	**
	(0.032)	(0.105)	(0.092)	
Jailed	−0.168	−0.375	−0.008	
	(0.077)	(0.218)	(0.031)	
Lost home to disaster	−0.002	0.006	−0.037	
	(0.020)	(0.095)	(0.036)	
No. of observations	100,300	10,973	10,719	21,692
No. of individuals	24,317	7592	7198	13,990

*Note:* Figures are coefficient estimates from three separate fixed‐effects (within‐person) models of hope by locus of control type. These regressions should be read vertically. External locus of control subsample (bottom 25% of the external‐internal locus of control scale) is presented in the second column. Internal locus of control subsample (top 25% of the external‐internal locus of control scale) is presented in the third column. The dependent hope variable ranges from 1 (totally hopeless) to 5 (totally hopeful). Standard errors clustered at the individual level are presented in parentheses. All reported life events are summarized in Table 3. Included in the hope equations but not shown are the covariates age, education, marital status, household composition, employment status, household income, neighborhood SES index, homeownership status, residential area, and year dummies. The average age in the full sample is 45.4 years (ranging from 15 to 101). Age distribution of respondents: 17% (15–24); 17% (25–34); 16% (35–44); 17% (45–54); 15% (55–64); 11% (65–74); 7% (≥ 75 years old). *p*‐values are corrected using the Bonferroni method (see Bland and Altman 1995). In this table, we multiply the original *p*‐values by 3; as we have 3 separate regression‐equations (columns)—across which we compare the estimated life‐event coefficients. Two‐sided *test of difference* is based on interaction terms from a fixed‐effects hope equation—using the subsample of external‐LOC and internal‐LOC individuals only. The estimated regression includes interaction terms between the negative life events and a binary indicator for whether or not the individual is in the external‐LOC group. * and ** denote statistical significance at the 5% and 1% levels, respectively.

Thus, in addition to hope in general, internal locus of control, which is correlated with hope, serves as an additional channel in both driving hope and serving as a protective mechanism as people navigate negative life events. While it is impossible at this juncture to know the direction of causality in the hope and internal locus of control relationship, the combination of the two seems to strengthen the positive effects of hope on future outcomes and the positive psychological buffer that is part of it.

### 3.5 Robustness to Sampling Issues

#### 3.5.1 Selective Attrition

A possible caveat in this kind of longitudinal analysis is selective attrition: respondents who have low levels of hope may be more likely to drop out of the sample. On the other hand, those with high levels of hope may be more likely to stay.

We find some evidence of such selective participation in Supporting Information S1: Table A15. Participation in the next wave was slightly lower when the respondent was a university or high‐school graduate; employed; from a high‐income household; recently lost a home due to a natural disaster; less satisfied with life; or felt less hopeful. The estimated coefficient on the high‐hope dummy variable is 0.015, suggesting that respondents with high levels of hope were 1.5 percentage points more likely to participate in the next survey wave than those with lower levels of hope. Thus, non‐random attrition of respondents biases our results slightly upwards. This is an important issue, especially for analyzing the future consequences of hope over the life course.

As a more formal exploration of this possible selection effect, we exclude the leavers or attritors ex ante by restricting our estimation sample to respondents who are present during the entire observation period: that is, in each of the eight survey waves from 2007 to 2021. This leaves us with a balanced panel of 5372 individuals and 42,976 observations. Supporting Information S1: Table A16 reports estimation results with the restricted sample. Here, we estimate the same prospective equations from Table 5—that calculate the effects of current hope on later life outcomes. In nearly all the life domains, the coefficient estimates are only slightly different.

For some outcomes and estimated slopes—such as getting a bachelor's degree; losing or changing jobs; being obese or heavy smoker—there is a loss of statistical significance, after we adjust for multiple hypothesis testing. Overall, though, most of the empirical results and qualitative conclusions are very similar to those found in the full analyzed sample, suggesting that selective attrition has some but little impact on our findings.

## 4 Conclusion

In this article, we provide the first (that we know of) large‐scale analysis showing the links between hope and a range of long‐term life outcomes, based on a large nationally representative longitudinal survey covering roughly 25,000 Australians for 14 years. We find a strong link between high levels of hope and better contemporary and future outcomes in the wellbeing, economic, health, and social arenas, based on both cross‐section and fixed effects specifications.

People with high levels of hope have higher levels of wellbeing, education, earning and employment outcomes, perceived and objective health indicators, and are much more likely to have friends and less likely to be lonely than those with low levels of hope. As these are all facets of life that are critical to quality of life and to longevity, we believe that better understanding the drivers of hope and its consequences can ultimately inform the ability of both individuals and of public policy to improve people's lives.

We explored some of the statistical channels through which hope operates—as a moderator—and leads to better outcomes. We identified these as resilience/psychological buffers, ability to adapt, and internal locus of control. We found that all of these are in play throughout the repeated waves of the HILDA data. People with high levels of hope were less likely to be affected by negative life events and they adapted more quickly and completely after those events. People with hope also had a higher internal locus of control, which was persistent through the 14‐year period we were able to study and was also, on its own, linked to better future outcomes.

The absence of hope in some population cohorts is also worthy of concern, as those without hope are likely to live lives that are shorter, sicker, and less prosperous. In the most extreme outcomes, such as the crisis of deaths of despair in the United States (premature mortality due to suicide, drug overdoses, and alcohol‐related diseases, the loss of hope (e.g., despair) pre‐dated the mortality trends and could have served as a warning sign. As such, trends such as the seeming shift in hope from younger to older cohorts in recent years (which show up clearly in the HILDA data) should be a wake‐up call to health and other policy practitioners of a cohort that is vulnerable to mental illness and other facets of despair.

### 4.1 Limitations

As with any work based on observational and survey data, there are possible response biases based on expectations (e.g. the different expectations that may affect the responses of the rich vs. the poor, or of deprived individuals compared more privileged ones) and cultural norms (Latin Americans, e.g., have a positive response bias in surveys of wellbeing, while Eastern Europeans have a negative one; see Graham and Nikolova 2015). Beyond our fixed effects analysis, which accounts for these biases at least in part, there is a limit to how much we can do.

Similarly, the issue of reverse causality is an important potential limitation. In the present study, we do not have exogenous variation in feelings of hope, that is, hope is not randomized across the studied sample of individuals. Neither are some of the other outcome variables such as life satisfaction and locus of control. Thus, we cannot prove whether hope causes people to be more satisfied with life, or whether higher life satisfaction causally leads people to be more hopeful. Even though our fixed‐effects models eliminate individual‐specific characteristics and traits that are unobserved and fixed over time, and which may simultaneously influence hope levels and the studied outcomes, we still need to understand more about the underlying causes and drivers of why people report higher or lower levels of hope.

Another issue is the extent to which people are hopeful because they can foresee or predict their futures (and of course, wealthier people are likely to be better informed), which would suggest that causality to better outcomes runs from having advantages such as income and education, rather than from hope to those outcomes. Yet our findings about things such as hope and future illnesses or the effects of hope playing more of a larger role in the education investments of the poor compared to the rich suggest there is more at play than the ability to foresee the future. The agentic properties of hope protect people in bad times and spur their continued efforts to improve their conditions in a way that raw optimism or future foresight would not.

We hope to take some of these issues on in future iterations of the work, for example exploring the drivers and consequences of hope in migrants versus non‐migrants or in the rich versus the poor in greater detail. We also plan to explore the difference in how people with high hope versus low hope are affected by and adapt to random shocks, such as a death in the family or a weather‐related disaster such as a lost home. As there are many non‐random shocks in the data, such as incarceration and financial losses, we can see if hope operates differently in situations that people can do nothing about versus those where strategic efforts can limit the damage.

## Conflicts of Interest

The authors declare no conflicts of interest.

## Supporting information


Supporting Information S1

## Data Availability

Data are from the Household, Income and Labor Dynamics in Australia (HILDA) Survey, with years from 2007 to 2021. This dataset is widely accessible for researchers, but we are not permitted to repost these datasets to a repository.
