# Supplementary material for: Hope and the Life Course: Results From a Longitudinal Study of 25,000 Adults
Source: Health Econ. 2025 Oct 7;35(1):90–101. doi: 10.1002/hec.70041 (PMC12680909; doi:10.1002/hec.70041)
Supplement: Supplementary file 1 — Supporting Information S1 [file HEC-35-90-s001.docx]

**Online Appendix**

**Hope and the Life Course: Results from a Longitudinal Study of 25,000 Adults**

**This supplementary online appendix contains the following sections:**

- Socioeconomic Determinants of Hope
- Figures A1-A6
- Tables A1-A17

**Socioeconomic Determinants of Hope**

In this supplementary section, we present complementary results on the determinants of hope from pooled OLS models without individual fixed effects. The estimated coefficients in Table A3 suggest that age, gender (being male), marriage, university education, formal employment, retirement, high income, neighborhood prosperity, homeownership, rural living, a healthy BMI, and strong social ties are all positively and strongly correlated with contemporaneous feelings of hope.

On the other hand, factors such as being a high-school dropout, not having a job, living in a major city, being a migrant, having poor health, being a heavy alcohol drinker, heavy cigarette smoker, obese, and feeling lonely are negatively correlated with hope. Additionally, negative events such as serious financial losses (e.g., bankruptcy), being fired or losing a job, getting divorced, death of a family member or close friend, serious injury or illness, being a victim of physical assault, going to jail, and losing a home to a natural disaster are linked with reduced levels of hope.

Intuitively, those with higher levels of education are more hopeful than those with less, and employed people are more hopeful than unemployed ones, neither of which is a surprise (Table A3). There are modest differences among those with lower levels of income compared to higher ones, with the latter being slightly more hopeful, but much greater difference between those who report poor health versus good health (in the expected direction). Similarly, the difference between those who report being lonely and those who do not is much larger than the income-driven gaps.

While there are almost no differences between those who did or did not have monetary gains in past years, there is indeed a significant gap for those who had major financial losses versus those who did not. This supports the theory that people value losses disproportionately to gains (Kahneman and Tversky 1979; De Neve et al. 2018). In the same vein, those with serious illnesses or injuries were far less hopeful than those who did not experience these, and those who were incarcerated were (not surprisingly) the least hopeful cohort. The largest negative coefficients on hope are self-reported poor health and being lonely, while the most positive ones are being above age 75, employed full-time, being married, and having friends.

While many of the same predictors of hope are also found in our panel data fixed-effects estimates in Table A4, a few of the socio-economic characteristics that are predictive of higher hope in the cross-section are not significant predictors across time within individuals. The results from the fixed-effects models show that neither a university degree, unemployment, nor homeownership are statistically significant predictors of hope. This comparison of the OLS and fixed-effects estimates (see Tables A3 and A4) suggests that some determinants of hope are steady over time.

Our regression results above of the determinants of hope are mostly intuitive, but there are some surprises. The first, as illustrated in Figure A1, is that hope increases in a monotonic manner with age as we follow the same individuals longitudinally over time (up to age 75 in the fixed effects models in Table A4), a change from earlier work comparing trends in life satisfaction to future life satisfaction, which serves as a proxy measure of optimism or hope (depending how it is asked). The earlier work found that while life satisfaction improves with age, optimism declines after the age 60, as even people with high life satisfaction understand that their futures are limited and start to experience declines in health (see Blanchflower and Graham 2022; Odermatt and Stutzer 2019). In some recent work on optimism among low-income cohorts in the U.S., meanwhile, optimism also declines beginning around age 60, except for African Americans, who are not only by far the most optimistic of the racial groups but also maintain their levels well into old age (see Graham and Pinto 2019).

We do not fully know what explains the change in the HILDA data, but it does accord with a significant drop in wellbeing and increase in mental health disorders among the young in recent years (in the US it began in 2011) that seems to have been exacerbated by COVID but it is linked to other factors – such as the increase in social media use by increasingly younger cohorts.

Supplementary Table A5 in the online appendix reports slope coefficients from cross-sectional and fixed effects hope equations that are estimated separately for men and women. Table A6 extends the same analysis by income level – for the rich and the poor.

There is some evidence of a gender difference when it comes to employment and feelings of hope in the same year (Table A5, columns 3 and 4): working males appear to experience steeper increases in hope than working females. While there are no obvious gender differences in hope for negative life events such as major financial losses, there does appear to be a differential response to losing a job or getting fired – with the fall in hope for women being more than twice in size than for men (with estimated coefficients of -0.070 and -0.025, respectively). On the other hand, men seem to be, on average, more sensitive to marital separation or divorce than women.

Not surprisingly, major financial setbacks are found to hurt the poor significantly more than the rich – as measured by contemporaneous changes in hope (Table A6, columns 3 and 4). A similar differential response by income group is also found for other life shocks – such as serious personal injury or illness – with poor individuals experiencing almost twice the fall in hope relative to rich individuals (with estimated slope coefficients of -0.11 and -0.06, respectively).

**Supplementary References**

Blanchflower, D. and Graham, C. (2022). The Mid‑Life Dip in Well‑Being: A Critique. *Social Indicators Research*, 161, 287-344.

De Neve, J. E., Ward, G., De Keulenaer, F., Van Landeghem, B., Kavetsos, G., & Norton, M. (2018). The asymmetric experience of positive and negative economic growth: Global evidence using subjective well-being data. *Review of Economics and Statistics*, 100(2), 362-375.

Graham, C. and Pinto, S. (2019). Unequal hopes and lives in the USA: optimism, race, place, and premature mortality. *Journal of Population Economics*, Vol.32 (2).

Kahneman, D. and Tversky, A. (1979). Prospect Theory: An Analysis of Decision under Risk. *Econometrica*, 47(2), 263–292.

Odermatt, R. and Stutzer, A. (2019). (Mis) Predicted Subjective Well-being Following Life Events, *Journal of the European Economics Association*, 17(1), 245-283.

|  | Mean | SD | Min | Max |
| --- | --- | --- | --- | --- |
| Age | 45.41 | 18.89 | 15 | 101 |
| Male | 0.47 | 0.50 | 0 | 1 |
| Aboriginal or TSI | 0.003 | 0.06 | 0 | 1 |
| Migrant | 0.21 | 0.40 | 0 | 1 |
| **Marital status** |  |  |  |  |
| Married | 0.48 | 0.50 | 0 | 1 |
| De-facto | 0.16 | 0.36 | 0 | 1 |
| Separated | 0.03 | 0.16 | 0 | 1 |
| Divorced | 0.06 | 0.24 | 0 | 1 |
| Widowed | 0.05 | 0.21 | 0 | 1 |
| Never married and not de-facto | 0.23 | 0.42 | 0 | 1 |
| **Household composition** |  |  |  |  |
| No. of children | 0.55 | 0.97 | 0 | 11 |
| No. of children (aged 0-4) | 0.19 | 0.51 | 0 | 4 |
| No. of children (aged 5-14) | 0.36 | 0.76 | 0 | 11 |
| No. of adults (aged 15+) | 2.31 | 1.04 | 1 | 10 |
| **Living status** |  |  |  |  |
| Homeowner | 0.69 | 0.46 | 0 | 1 |
| Lives in major city | 0.62 | 0.49 | 0 | 1 |
| Lives in inner regional area | 0.26 | 0.44 | 0 | 1 |
| Lives in rural area | 0.12 | 0.33 | 0 | 1 |
| **Education level** |  |  |  |  |
| University degree | 0.26 | 0.44 | 0 | 1 |
| Postgraduate degree | 0.05 | 0.22 | 0 | 1 |
| Graduate diploma | 0.06 | 0.23 | 0 | 1 |
| Bachelor’s degree | 0.15 | 0.35 | 0 | 1 |
| Diploma | 0.09 | 0.29 | 0 | 1 |
| Certificate (any level) | 0.22 | 0.41 | 0 | 1 |
| High-school graduate | 0.15 | 0.36 | 0 | 1 |
| High-school drop-out | 0.28 | 0.45 | 0 | 1 |
| **Employment status** |  |  |  |  |
| Employed FT or PT | 0.63 | 0.48 | 0 | 1 |
| Employed FT | 0.42 | 0.49 | 0 | 1 |
| Employed PT | 0.22 | 0.41 | 0 | 1 |
| Self-employed | 0.09 | 0.29 | 0 | 1 |
| Unemployed | 0.04 | 0.19 | 0 | 1 |
| Not in labor force | 0.33 | 0.47 | 0 | 1 |
| Retired | 0.18 | 0.38 | 0 | 1 |
| **Income and SES** |  |  |  |  |
| Work hours per week | 35.17 | 14.75 | 1 | 140 |
| HH disposable income | 95,361 | 71,718 | 0 | 973,627 |
| Log of HH income | 11.23 | 0.76 | 3.22 | 13.79 |
| Neighborhood SES index | 5.53 | 2.87 | 1 | 10 |

SUPPLEMENTARY SUMMARY TABLES

(Tables A1-A2)

Table A1. Summary of Demographic and Socioeconomic Variables, HILDA Survey 2007-2021

Table A2. Summary of Health Status and Social Life Variables, HILDA Survey 2007-2021

|  | Mean | SD | Min | Max |
| --- | --- | --- | --- | --- |
| **Health status**  Long-term health issue | 0.25 | 0.43 | 0 | 1 |
| Poor health (self-reported) | 0.17 | 0.38 | 0 | 1 |
| Smoke cigarettes | 0.45 | 0.50 | 0 | 1 |
| Smoke cigarettes every day | 0.14 | 0.35 | 0 | 1 |
| Drink alcohol | 0.81 | 0.39 | 0 | 1 |
| Drink alcohol almost every day | 0.14 | 0.35 | 0 | 1 |
| Hospital admissions in past year | 0.20 | 0.78 | 0 | 50 |
| Body Mass Index (BMI) | 26.93 | 5.78 | 11.4 | 91.5 |
| BMI: Underweight | 0.02 | 0.15 | 0 | 1 |
| BMI: Healthy Weight | 0.37 | 0.48 | 0 | 1 |
| BMI: Overweight | 0.33 | 0.47 | 0 | 1 |
| BMI: Obese | 0.28 | 0.45 | 0 | 1 |
| **Social life** |  |  |  |  |
| Meet friends almost every day | 0.26 | 0.44 | 0 | 1 |
| Meet friends every 3 months | 0.12 | 0.32 | 0 | 1 |
| People don’t visit me | 3.50 | 1.84 | 1 | 7 |
| Don’t get much help in life | 2.31 | 1.59 | 1 | 7 |
| Have lots of friends | 4.40 | 1.70 | 1 | 7 |
| Have no one to confide in | 2.33 | 1.70 | 1 | 7 |
| Have no one to lean on | 2.19 | 1.63 | 1 | 7 |
| Have someone to cheer me up | 5.35 | 1.69 | 1 | 7 |
| Feel very lonely | 2.64 | 1.78 | 1 | 7 |

SUPPLEMENTARY FIGURES

(Figures A1-A6)

Figure A1. Feelings of hope over the lifespan, HILDA Survey 2007-2021

*Notes:* The figure plots the predicted within-person feelings of hope over age. The plot is derived from the estimated coefficients from a fixed-effects model of hope with eight age-band dummies. Included in the hope regression are the covariates marital status, household composition, education, employment status, household income, neighborhood SES, homeownership status, geographical indicators, and year. Standard errors clustered at the individual level. The hope measure ranges from 1 (totally hopeless) to 5 (totally hopeful). Sample consists of 25,713 individuals and 115,578 person-year observations.

Figure A2. A Kernel plot of the standardized locus of control distribution

[FIGURE A3]

1. **Major improvement in finances**
2. **Major worsening in finances**

Figure A3. Leads and Lags in Hope to Major Life Events, HILDA Survey 2007-2021

*Notes:* Each value on the vertical y-axis represents the lead and lag coefficients of the major life events as reported in Table A7. The life event of interest took place at year *t=0*. Periods *t-1* and *t-2* capture 2-3 years and 4-5 years before or leading up to the event, respectively. Periods *t+1* and *t+2* capture 2-3 years and 4-5 years after or following the event, respectively. All reported life events are summarized in Table 3. Number of individuals and person-year observations equals 10,284 and 24,089 respectively. Vertical lines represent 95% confidence intervals at each time period.

Figure A3 *continued.*

1. **Promoted**
2. **Fired or made redundant**

Figure A3 *continued.*

1. **Married**
2. **Separated from spouse**

Figure A3 *continued.*

1. **Pregnancy**
2. **Birth of child**

Figure A3 *continued.*

1. **Death of spouse or child**
2. **Serious personal injury or illness**

Figure A3 *continued.*

1. **Victim of physical violence**
2. **Jailed**

Figure A3 *continued.*

1. **Lost home to natural disaster**

[FIGURE A4]

Males Females

1. **Major improvement in finances**

Males Females

1. **Major worsening in finances**

Figure A4. Leads and Lags in Hope to Major Life Events by Gender, HILDA Survey 2007-2021

*Notes:* Each value on the vertical y-axis represents the lead and lag coefficients of the major life events as reported in Table A8. The life event of interest took place at year *t=0*. Periods *t-1* and *t-2* capture 2-3 years and 4-5 years before or leading up to the event, respectively. Periods *t+1* and *t+2* capture 2-3 years and 4-5 years after or following the event, respectively. All reported life events are summarised in Table 3. Number of person-year observations equals 13,126 for females and 10,963 for males. Vertical lines represent 95% confidence intervals at each time period.

Figure A4 *continued.*

Males Females

1. **Promoted**

Males Females

1. **Fired or made redundant**

Figure A4 *continued.*

Males Females

1. **Married**

Males Females

1. **Separated from spouse**

Figure A4 *continued.*

Males Females

1. **Pregnancy**

Males Females

1. **Birth of child**

Figure A4 *continued.*

Males Females

1. **Death of spouse or child**

Males Females

1. **Serious personal injury or illness**

Figure A4 *continued.*

Males Females

1. **Victim of physical violence**

Males Females

1. **Jailed**

Figure A4 *continued.*

Males Females

1. **Lost home to natural disaster**

[FIGURE A5]

Poor Rich

1. **Major improvement in finances**

Poor Rich

1. **Major worsening in finances**

Figure A5. Leads and Lags in Hope to Major Life Events by Income, HILDA Survey 2007-2021

*Notes:* Each value on the vertical y-axis represents the lead and lag coefficients of the major life events as reported in Table A9. The life event of interest took place at year *t=0*. Periods *t-1* and *t-2* capture 2-3 years and 4-5 years before or leading up to the event, respectively. Periods *t+1* and *t+2* capture 2-3 years and 4-5 years after or following the event, respectively. Poor subsample captures individuals in the bottom income quartile. Rich subsample captures individuals in the top income quartile. All reported life events are summarized in Table 3. The number of person-year observations equals 5,585 for poor subsample and 6,448 for rich subsample. Vertical lines represent 95% confidence intervals at each time period.

Figure A5 *continued.*

Poor Rich

1. **Promoted**

Poor Rich

1. **Fired or made redundant**

Figure A5 *continued.*

Poor Rich

1. **Married**

Poor Rich

1. **Separated from spouse**

Figure A5 *continued.*

Poor Rich

1. **Pregnancy**

Poor Rich

1. **Birth of child**

Figure A5 *continued.*

Poor Rich

1. **Death of spouse or child**

Poor Rich

1. **Serious personal injury or illness**

Figure A5 *continued.*

Poor Rich

1. **Victim of physical violence**

Poor Rich

1. **Jailed**

Figure A5 *continued.*

Poor Rich

1. **Lost home to natural disaster**

[FIGURE A6]

External LOC (bottom 25%) Internal LOC (top 25%)

1. **Major worsening in finances**

External LOC (bottom 25%) Internal LOC (top 25%)

1. **Fired or made redundant**

Figure A6. Leads and Lags in Hope to Negative Life Events by Locus of Conrol Type, HILDA Survey

*Notes:* Each value on the vertical y-axis represents the lead and lag coefficients of the major life events as reported in Table A12. External locus of control subsample captures individuals in the bottom 25% of the external-internal locus of control scale. Internal locus of control subsample captures individuals in the top 25% of the external-internal locus of control scale. The locus of control scale is summarized in Table 2. Locus of control questions are available in the HILDA data for years 2007, 2011, 2015, and 2019 only. The life event of interest took place at year *t=0*. Period *t-1* captures 3-4 years before or leading up to the event. Period *t+1* captures 2-3 years after or following the event. All reported life events are summarized in Table 3. The number of person-year observations equals 7,206 for external LOC subsample and 7,599 for internal LOC subsample. Vertical lines represent 95% confidence intervals at each time period.

Figure A6 *continued.*

External LOC (bottom 25%) Internal LOC (top 25%)

1. **Separated from spouse**

External LOC (bottom 25%) Internal LOC (top 25%)

1. **Death of spouse or child**

Figure A6 *continued.*

External LOC (bottom 25%) Internal LOC (top 25%)

1. **Serious personal injury or illness**

External LOC (bottom 25%) Internal LOC (top 25%)

1. **Victim of physical violence**

Figure A6 *continued.*

External LOC (bottom 25%) Internal LOC (top 25%)

1. **Jailed**

External LOC (bottom 25%) Internal LOC (top 25%)

1. **Lost home to natural disaster**

SUPPLEMENTARY REGRESSION TABLES

(Tables A3-A17)

Table A3. Determinants of Hope from Cross-Sectional Regression Models, HILDA Survey 2007-2021

|  | (1) | | (2) | | (3) | | (4) | | (5) | |
| --- | --- | --- | --- | --- | --- | --- | --- | --- | --- | --- |
| Male | 0.099 | ** | 0.057 | ** | 0.074 | ** | 0.069 | ** | 0.065 | ** |
|  | (0.008) |  | (0.008) |  | (0.008) |  | (0.007) |  | (0.008) |  |
| **Age group** |  |  |  |  |  |  |  |  |  |  |
| 25-34 | 0.120 | ** | -0.050 | ** | 0.014 |  | 0.036 | ** | -0.042 | ** |
|  | (0.011) |  | (0.014) |  | (0.013) |  | (0.012) |  | (0.015) |  |
| 35-44 | 0.196 | ** | -0.034 | * | 0.075 | ** | 0.089 | ** | -0.009 |  |
|  | (0.013) |  | (0.017) |  | (0.016) |  | (0.014) |  | (0.018) |  |
| 45-54 | 0.240 | ** | 0.026 |  | 0.159 | ** | 0.163 | ** | 0.068 | ** |
|  | (0.013) |  | (0.016) |  | (0.015) |  | (0.013) |  | (0.017) |  |
| 55-64 | 0.323 | ** | 0.145 | ** | 0.289 | ** | 0.240 | ** | 0.177 | ** |
|  | (0.013) |  | (0.017) |  | (0.016) |  | (0.014) |  | (0.018) |  |
| 65-74 | 0.431 | ** | 0.314 | ** | 0.437 | ** | 0.339 | ** | 0.335 | ** |
|  | (0.013) |  | (0.019) |  | (0.018) |  | (0.016) |  | (0.020) |  |
| 75-84 | 0.433 | ** | 0.352 | ** | 0.501 | ** | 0.377 | ** | 0.379 | ** |
|  | (0.015) |  | (0.022) |  | (0.021) |  | (0.019) |  | (0.023) |  |
| 85+ | 0.398 | ** | 0.344 | ** | 0.530 | ** | 0.380 | ** | 0.363 | ** |
|  | (0.022) |  | (0.030) |  | (0.028) |  | (0.026) |  | (0.031) |  |
| **Marital status** |  |  |  |  |  |  |  |  |  |  |
| Married |  |  | 0.162 | ** | 0.136 | ** | 0.054 | ** | 0.140 | ** |
|  |  |  | (0.014) |  | (0.013) |  | (0.012) |  | (0.015) |  |
| De-facto |  |  | 0.078 | ** | 0.072 | ** | 0.005 |  | 0.071 | ** |
|  |  |  | (0.013) |  | (0.013) |  | (0.011) |  | (0.014) |  |
| Separated |  |  | -0.066 | * | -0.051 | * | -0.025 |  | 0.003 |  |
|  |  |  | (0.028) |  | (0.026) |  | (0.023) |  | (0.028) |  |
| Divorced |  |  | 0.025 |  | 0.044 | * | 0.041 | * | 0.042 |  |
|  |  |  | (0.022) |  | (0.021) |  | (0.018) |  | (0.022) |  |
| Widowed |  |  | 0.094 | ** | 0.094 | ** | 0.116 | ** | 0.117 | ** |
|  |  |  | (0.024) |  | (0.022) |  | (0.020) |  | (0.025) |  |
| **Household composition** |  |  |  |  |  |  |  |  |  |  |
| No. of children |  |  | 0.012 | ** | 0.004 |  | 0.021 | ** | 0.007 |  |
|  |  |  | (0.004) |  | (0.004) |  | (0.004) |  | (0.005) |  |
| No. of adults |  |  | -0.027 | ** | -0.019 | ** | -0.023 | ** | -0.023 | ** |
|  |  |  | (0.004) |  | (0.004) |  | (0.003) |  | (0.004) |  |
| **Education level** |  |  |  |  |  |  |  |  |  |  |
| University degree |  |  | 0.075 | ** | 0.032 | ** | 0.030 | ** | 0.075 | ** |
|  |  |  | (0.012) |  | (0.011) |  | (0.010) |  | (0.012) |  |
| Diploma/certificate |  |  | 0.025 | * | 0.013 |  | 0.022 | * | 0.036 | ** |
|  |  |  | (0.011) |  | (0.010) |  | (0.009) |  | (0.011) |  |
| High school graduate |  |  | 0.040 | ** | 0.021 |  | 0.016 |  | 0.038 | ** |
|  |  |  | (0.013) |  | (0.012) |  | (0.011) |  | (0.013) |  |
| **Employment status** |  |  |  |  |  |  |  |  |  |  |
| Employed full-time |  |  | 0.303 | ** | 0.196 | ** | 0.203 | ** | 0.260 | ** |
|  |  |  | (0.013) |  | (0.012) |  | (0.011) |  | (0.014) |  |
| Employed part-time |  |  | 0.239 | ** | 0.148 | ** | 0.156 | ** | 0.211 | ** |
|  |  |  | (0.012) |  | (0.011) |  | (0.010) |  | (0.013) |  |
| Unemployed |  |  | -0.063 | ** | -0.109 | ** | -0.036 | * | -0.018 |  |
|  |  |  | (0.020) |  | (0.019) |  | (0.017) |  | (0.021) |  |
| Retired |  |  | 0.177 | ** | 0.150 | ** | 0.103 | ** | 0.148 | ** |
|  |  |  | (0.014) |  | (0.013) |  | (0.012) |  | (0.015) |  |
| **Income quartile** |  |  |  |  |  |  |  |  |  |  |
| 2nd quartile |  |  | 0.082 | ** |  |  |  |  |  |  |
|  |  |  | (0.010) |  |  |  |  |  |  |  |
| 3rd quartile |  |  | 0.119 | ** |  |  |  |  |  |  |
|  |  |  | (0.011) |  |  |  |  |  |  |  |
| Highest quartile |  |  | 0.164 | ** |  |  |  |  |  |  |
|  |  |  | (0.012) |  |  |  |  |  |  |  |
| **Neighborhood SES** |  |  |  |  |  |  |  |  |  |  |
| 2nd decile |  |  | 0.069 | ** |  |  |  |  |  |  |
|  |  |  | (0.018) |  |  |  |  |  |  |  |
| 3rd decile |  |  | 0.105 | ** |  |  |  |  |  |  |
|  |  |  | (0.017) |  |  |  |  |  |  |  |
| 4th decile |  |  | 0.137 | ** |  |  |  |  |  |  |
|  |  |  | (0.017) |  |  |  |  |  |  |  |
| 5th decile |  |  | 0.111 | ** |  |  |  |  |  |  |
|  |  |  | (0.017) |  |  |  |  |  |  |  |
| 6th decile |  |  | 0.131 | ** |  |  |  |  |  |  |
|  |  |  | (0.018) |  |  |  |  |  |  |  |
| 7th decile |  |  | 0.120 | ** |  |  |  |  |  |  |
|  |  |  | (0.018) |  |  |  |  |  |  |  |
| 8th decile |  |  | 0.158 | ** |  |  |  |  |  |  |
|  |  |  | (0.017) |  |  |  |  |  |  |  |
| 9th decile |  |  | 0.172 | ** |  |  |  |  |  |  |
|  |  |  | (0.017) |  |  |  |  |  |  |  |
| Highest decile |  |  | 0.170 | ** |  |  |  |  |  |  |
|  |  |  | (0.018) |  |  |  |  |  |  |  |
| **Living status** |  |  |  |  |  |  |  |  |  |  |
| Homeowner |  |  | 0.113 | ** | 0.074 | ** | 0.066 | ** | 0.094 | ** |
|  |  |  | (0.009) |  | (0.008) |  | (0.007) |  | (0.009) |  |
| Live in major city |  |  | -0.058 | ** | -0.054 | ** | -0.043 | ** | -0.054 | ** |
|  |  |  | (0.012) |  | (0.012) |  | (0.010) |  | (0.013) |  |
| Live in inner regional |  |  | -0.031 | * | -0.033 | ** | -0.018 |  | -0.022 |  |
|  |  |  | (0.013) |  | (0.012) |  | (0.011) |  | (0.013) |  |
| Migrant |  |  | -0.039 | ** | -0.050 | ** | -0.017 | * | -0.044 | ** |
|  |  |  | (0.010) |  | (0.009) |  | (0.008) |  | (0.010) |  |
| **Household income** |  |  |  |  |  |  |  |  |  |  |
| Log of HH income |  |  |  |  | 0.052 | ** | 0.039 | ** | 0.065 | ** |
|  |  |  |  |  | (0.005) |  | (0.005) |  | (0.006) |  |
| Neighborhood SES |  |  |  |  | 0.006 | ** | 0.004 | ** | 0.014 | ** |
|  |  |  |  |  | (0.001) |  | (0.001) |  | (0.001) |  |
| **Health status** |  |  |  |  |  |  |  |  |  |  |
| Poor health |  |  |  |  | -0.529 | ** |  |  |  |  |
|  |  |  |  |  | (0.011) |  |  |  |  |  |
| Long-term health issue |  |  |  |  | -0.107 | ** |  |  |  |  |
|  |  |  |  |  | (0.008) |  |  |  |  |  |
| Smoke every day |  |  |  |  | -0.134 | ** |  |  |  |  |
|  |  |  |  |  | (0.011) |  |  |  |  |  |
| Drink alcohol every day |  |  |  |  | -0.030 | ** |  |  |  |  |
|  |  |  |  |  | (0.008) |  |  |  |  |  |
| BMI: Underweight |  |  |  |  | 0.027 |  |  |  |  |  |
|  |  |  |  |  | (0.023) |  |  |  |  |  |
| BMI: Healthy Weight |  |  |  |  | 0.069 | ** |  |  |  |  |
|  |  |  |  |  | (0.009) |  |  |  |  |  |
| BMI: Overweight |  |  |  |  | 0.038 | ** |  |  |  |  |
|  |  |  |  |  | (0.008) |  |  |  |  |  |
| **Social life** |  |  |  |  |  |  |  |  |  |  |
| Meet with friends almost every day |  |  |  |  |  |  | 0.027 | ** |  |  |
|  |  |  |  |  |  |  | (0.006) |  |  |  |
| Have lots of friends |  |  |  |  |  |  | 0.039 | ** |  |  |
|  |  |  |  |  |  |  | (0.002) |  |  |  |
| Don’t get much help |  |  |  |  |  |  | -0.073 | ** |  |  |
|  |  |  |  |  |  |  | (0.002) |  |  |  |
| No one to lean on |  |  |  |  |  |  | -0.030 | ** |  |  |
|  |  |  |  |  |  |  | (0.002) |  |  |  |
| Feel very lonely |  |  |  |  |  |  | -0.141 | ** |  |  |
|  |  |  |  |  |  |  | (0.002) |  |  |  |
| **Major life events** |  |  |  |  |  |  |  |  |  |  |
| Money gained |  |  |  |  |  |  |  |  | 0.036 | * |
|  |  |  |  |  |  |  |  |  | (0.015) |  |
| Money lost |  |  |  |  |  |  |  |  | -0.480 | ** |
|  |  |  |  |  |  |  |  |  | (0.024) |  |
| Hired |  |  |  |  |  |  |  |  | 0.003 |  |
|  |  |  |  |  |  |  |  |  | (0.009) |  |
| Fired |  |  |  |  |  |  |  |  | -0.089 | ** |
|  |  |  |  |  |  |  |  |  | (0.018) |  |
| Promoted |  |  |  |  |  |  |  |  | 0.050 | ** |
|  |  |  |  |  |  |  |  |  | (0.010) |  |
| Retired |  |  |  |  |  |  |  |  | -0.009 |  |
|  |  |  |  |  |  |  |  |  | (0.019) |  |
| Married |  |  |  |  |  |  |  |  | -0.008 |  |
|  |  |  |  |  |  |  |  |  | (0.019) |  |
| Pregnant |  |  |  |  |  |  |  |  | 0.079 | ** |
|  |  |  |  |  |  |  |  |  | (0.014) |  |
| Childbirth |  |  |  |  |  |  |  |  | 0.009 |  |
|  |  |  |  |  |  |  |  |  | (0.017) |  |
| Separated |  |  |  |  |  |  |  |  | -0.196 | ** |
|  |  |  |  |  |  |  |  |  | (0.019) |  |
| Reconciled |  |  |  |  |  |  |  |  | -0.136 | ** |
|  |  |  |  |  |  |  |  |  | (0.036) |  |
| Moved |  |  |  |  |  |  |  |  | 0.001 |  |
|  |  |  |  |  |  |  |  |  | (0.008) |  |
| Spouse/child died |  |  |  |  |  |  |  |  | -0.246 | ** |
|  |  |  |  |  |  |  |  |  | (0.040) |  |
| Friend died |  |  |  |  |  |  |  |  | -0.035 | ** |
|  |  |  |  |  |  |  |  |  | (0.009) |  |
| Relative died |  |  |  |  |  |  |  |  | -0.043 | ** |
|  |  |  |  |  |  |  |  |  | (0.009) |  |
| Health shock |  |  |  |  |  |  |  |  | -0.242 | ** |
|  |  |  |  |  |  |  |  |  | (0.012) |  |
| Physically attacked |  |  |  |  |  |  |  |  | -0.414 | ** |
|  |  |  |  |  |  |  |  |  | (0.034) |  |
| Family harmed |  |  |  |  |  |  |  |  | -0.044 | ** |
|  |  |  |  |  |  |  |  |  | (0.008) |  |
| Jailed |  |  |  |  |  |  |  |  | -0.228 | ** |
|  |  |  |  |  |  |  |  |  | (0.075) |  |
| Relative jailed |  |  |  |  |  |  |  |  | -0.054 | * |
|  |  |  |  |  |  |  |  |  | (0.026) |  |
| Home lost |  |  |  |  |  |  |  |  | -0.093 | ** |
|  |  |  |  |  |  |  |  |  | (0.024) |  |
| Intercept | 4.340 | ** | 4.011 | ** | 3.707 | ** | 4.240 | ** | 3.496 | ** |
|  | (0.011) |  | (0.025) |  | (0.057) |  | (0.054) |  | (0.064) |  |
| No. of observations | 115630 |  | 115577 |  | 113951 |  | 113474 |  | 100873 |  |
| Adjusted R-squared | 0.04 |  | 0.10 |  | 0.16 |  | 0.29 |  | 0.12 |  |
| *Notes:* Figures are estimated coefficients from cross-sectional pooled OLS models of hope. Robust standard errors clustered at the individual level are presented in parentheses. The estimates are based on the HILDA Survey data for years 2007, 2009, 2011, 2013, 2015, 2017, 2019, and 2021. The dependent hope variable ranges from 1 (totally hopeless) to 5 (totally hopeful). All included covariates are summarized in Table 3 and Supplementary Tables A1 and A2. Included in all regressions but not shown are survey year dummies. * and ** denote statistical significance at the 5% and 1% levels, respectively. | | | | | | | | | | |

Table A4. Determinants of Hope from Fixed-Effects Regression Models, HILDA Survey 2007-2021

|  | (1) | | (2) | | (3) | | (4) | | (5) | |
| --- | --- | --- | --- | --- | --- | --- | --- | --- | --- | --- |
| **Age group** |  |  |  |  |  |  |  |  |  |  |
| 25-34 | 0.001 |  | -0.037 | * | -0.042 | ** | -0.019 |  | -0.037 | * |
|  | (0.013) |  | (0.015) |  | (0.015) |  | (0.014) |  | (0.016) |  |
| 35-44 | -0.009 |  | -0.055 | ** | -0.063 | ** | -0.028 |  | -0.055 | * |
|  | (0.019) |  | (0.020) |  | (0.020) |  | (0.019) |  | (0.022) |  |
| 45-54 | 0.013 |  | -0.030 |  | -0.043 |  | -0.010 |  | -0.022 |  |
|  | (0.024) |  | (0.025) |  | (0.025) |  | (0.024) |  | (0.027) |  |
| 55-64 | 0.077 | ** | 0.045 |  | 0.030 |  | 0.039 |  | 0.054 |  |
|  | (0.029) |  | (0.030) |  | (0.030) |  | (0.029) |  | (0.032) |  |
| 65-74 | 0.178 | ** | 0.157 | ** | 0.138 | ** | 0.115 | ** | 0.154 | ** |
|  | (0.035) |  | (0.035) |  | (0.034) |  | (0.034) |  | (0.037) |  |
| 75-84 | 0.202 | ** | 0.188 | ** | 0.185 | ** | 0.138 | ** | 0.192 | ** |
|  | (0.040) |  | (0.040) |  | (0.040) |  | (0.039) |  | (0.043) |  |
| 85+ | 0.163 | ** | 0.159 | ** | 0.184 | ** | 0.124 | ** | 0.163 | ** |
|  | (0.048) |  | (0.048) |  | (0.048) |  | (0.048) |  | (0.051) |  |
| **Marital status** |  |  |  |  |  |  |  |  |  |  |
| Married |  |  | 0.090 | ** | 0.089 | ** | 0.068 | ** | 0.034 |  |
|  |  |  | (0.017) |  | (0.017) |  | (0.016) |  | (0.019) |  |
| De-facto |  |  | 0.085 | ** | 0.084 | ** | 0.046 | ** | 0.049 | ** |
|  |  |  | (0.014) |  | (0.014) |  | (0.013) |  | (0.015) |  |
| Separated |  |  | -0.071 | * | -0.064 | * | -0.023 |  | -0.039 |  |
|  |  |  | (0.028) |  | (0.028) |  | (0.026) |  | (0.030) |  |
| Divorced |  |  | 0.052 |  | 0.053 | * | 0.080 | ** | 0.065 | * |
|  |  |  | (0.027) |  | (0.026) |  | (0.025) |  | (0.030) |  |
| Widowed |  |  | 0.016 |  | 0.008 |  | 0.046 |  | 0.009 |  |
|  |  |  | (0.030) |  | (0.030) |  | (0.030) |  | (0.035) |  |
| **Household composition** |  |  |  |  |  |  |  |  |  |  |
| No. of children |  |  | 0.004 |  | 0.003 |  | 0.011 | * | 0.004 |  |
|  |  |  | (0.005) |  | (0.004) |  | (0.004) |  | (0.005) |  |
| No. of adults |  |  | -0.002 |  | -0.001 |  | -0.002 |  | -0.001 |  |
|  |  |  | (0.004) |  | (0.004) |  | (0.004) |  | (0.004) |  |
| **Education level** |  |  |  |  |  |  |  |  |  |  |
| University degree |  |  | -0.044 |  | -0.043 |  | -0.007 |  | -0.059 | * |
|  |  |  | (0.024) |  | (0.024) |  | (0.023) |  | (0.027) |  |
| Diploma/certificate |  |  | -0.039 |  | -0.039 |  | 0.005 |  | -0.045 | * |
|  |  |  | (0.020) |  | (0.020) |  | (0.019) |  | (0.023) |  |
| High school graduate |  |  | -0.062 | ** | -0.059 | ** | -0.022 |  | -0.069 | ** |
|  |  |  | (0.017) |  | (0.017) |  | (0.016) |  | (0.019) |  |
| **Employment status** |  |  |  |  |  |  |  |  |  |  |
| Employed full-time |  |  | 0.109 | ** | 0.090 | ** | 0.096 | ** | 0.077 | ** |
|  |  |  | (0.011) |  | (0.011) |  | (0.011) |  | (0.012) |  |
| Employed part-time |  |  | 0.072 | ** | 0.060 | ** | 0.062 | ** | 0.056 | ** |
|  |  |  | (0.010) |  | (0.010) |  | (0.010) |  | (0.011) |  |
| Unemployed |  |  | -0.032 |  | -0.046 | * | -0.027 |  | -0.021 |  |
|  |  |  | (0.018) |  | (0.018) |  | (0.017) |  | (0.020) |  |
| Retired |  |  | 0.066 | ** | 0.062 | ** | 0.047 | ** | 0.065 | ** |
|  |  |  | (0.011) |  | (0.011) |  | (0.011) |  | (0.012) |  |
| **Income quartile** |  |  |  |  |  |  |  |  |  |  |
| 2nd quartile |  |  | 0.026 | ** |  |  |  |  |  |  |
|  |  |  | (0.008) |  |  |  |  |  |  |  |
| 3rd quartile |  |  | 0.030 | ** |  |  |  |  |  |  |
|  |  |  | (0.009) |  |  |  |  |  |  |  |
| Highest quartile |  |  | 0.035 | ** |  |  |  |  |  |  |
|  |  |  | (0.011) |  |  |  |  |  |  |  |
| **Neighborhood SES** |  |  |  |  |  |  |  |  |  |  |
| 2nd decile |  |  | 0.011 |  |  |  |  |  |  |  |
|  |  |  | (0.019) |  |  |  |  |  |  |  |
| 3rd decile |  |  | 0.001 |  |  |  |  |  |  |  |
|  |  |  | (0.019) |  |  |  |  |  |  |  |
| 4th decile |  |  | 0.044 | * |  |  |  |  |  |  |
|  |  |  | (0.020) |  |  |  |  |  |  |  |
| 5th decile |  |  | 0.031 |  |  |  |  |  |  |  |
|  |  |  | (0.020) |  |  |  |  |  |  |  |
| 6th decile |  |  | 0.021 |  |  |  |  |  |  |  |
|  |  |  | (0.021) |  |  |  |  |  |  |  |
| 7th decile |  |  | 0.017 |  |  |  |  |  |  |  |
|  |  |  | (0.020) |  |  |  |  |  |  |  |
| 8th decile |  |  | 0.047 | * |  |  |  |  |  |  |
|  |  |  | (0.021) |  |  |  |  |  |  |  |
| 9th decile |  |  | 0.049 | * |  |  |  |  |  |  |
|  |  |  | (0.021) |  |  |  |  |  |  |  |
| Highest decile |  |  | 0.056 | * |  |  |  |  |  |  |
|  |  |  | (0.022) |  |  |  |  |  |  |  |
| **Living status** |  |  |  |  |  |  |  |  |  |  |
| Homeowner |  |  | 0.015 |  | 0.012 |  | 0.005 |  | 0.008 |  |
|  |  |  | (0.009) |  | (0.009) |  | (0.008) |  | (0.010) |  |
| Lives in major city |  |  | -0.050 | * | -0.052 | ** | -0.049 | ** | -0.056 | * |
|  |  |  | (0.020) |  | (0.020) |  | (0.018) |  | (0.022) |  |
| Lives in inner regional |  |  | -0.022 |  | -0.027 |  | -0.024 |  | -0.031 |  |
|  |  |  | (0.020) |  | (0.019) |  | (0.018) |  | (0.022) |  |
| **Household income** |  |  |  |  |  |  |  |  |  |  |
| Log of HH income |  |  |  |  | 0.016 | ** | 0.015 | ** | 0.012 | * |
|  |  |  |  |  | (0.005) |  | (0.005) |  | (0.006) |  |
| Neighborhood SES |  |  |  |  | 0.005 | * | 0.005 | ** | 0.005 | * |
|  |  |  |  |  | (0.002) |  | (0.002) |  | (0.002) |  |
| **Health status** |  |  |  |  |  |  |  |  |  |  |
| Poor health |  |  |  |  | -0.283 | ** |  |  |  |  |
|  |  |  |  |  | (0.010) |  |  |  |  |  |
| Long-term health issue |  |  |  |  | -0.040 | ** |  |  |  |  |
|  |  |  |  |  | (0.007) |  |  |  |  |  |
| Smoke every day |  |  |  |  | -0.029 | * |  |  |  |  |
|  |  |  |  |  | (0.014) |  |  |  |  |  |
| Drink alcohol every day |  |  |  |  | -0.045 | ** |  |  |  |  |
|  |  |  |  |  | (0.009) |  |  |  |  |  |
| BMI: Underweight |  |  |  |  | -0.011 |  |  |  |  |  |
|  |  |  |  |  | (0.024) |  |  |  |  |  |
| BMI: Healthy Weight |  |  |  |  | 0.014 |  |  |  |  |  |
|  |  |  |  |  | (0.011) |  |  |  |  |  |
| BMI: Overweight |  |  |  |  | 0.022 | * |  |  |  |  |
|  |  |  |  |  | (0.009) |  |  |  |  |  |
| **Social life** |  |  |  |  |  |  |  |  |  |  |
| Meet with friends almost every day |  |  |  |  |  |  | 0.033 | ** |  |  |
|  |  |  |  |  |  |  | (0.006) |  |  |  |
| Have lots of friends |  |  |  |  |  |  | 0.040 | ** |  |  |
|  |  |  |  |  |  |  | (0.002) |  |  |  |
| Don’t get much help |  |  |  |  |  |  | -0.045 | ** |  |  |
|  |  |  |  |  |  |  | (0.002) |  |  |  |
| No one to lean on |  |  |  |  |  |  | -0.023 | ** |  |  |
|  |  |  |  |  |  |  | (0.002) |  |  |  |
| Feel very lonely |  |  |  |  |  |  | -0.089 | ** |  |  |
|  |  |  |  |  |  |  | (0.002) |  |  |  |
| **Major life events** |  |  |  |  |  |  |  |  |  |  |
| Money gained |  |  |  |  |  |  |  |  | 0.014 |  |
|  |  |  |  |  |  |  |  |  | (0.013) |  |
| Money lost |  |  |  |  |  |  |  |  | -0.293 | ** |
|  |  |  |  |  |  |  |  |  | (0.020) |  |
| Hired |  |  |  |  |  |  |  |  | 0.024 | ** |
|  |  |  |  |  |  |  |  |  | (0.008) |  |
| Fired |  |  |  |  |  |  |  |  | -0.046 | ** |
|  |  |  |  |  |  |  |  |  | (0.016) |  |
| Promoted |  |  |  |  |  |  |  |  | 0.044 | ** |
|  |  |  |  |  |  |  |  |  | (0.010) |  |
| Retired |  |  |  |  |  |  |  |  | -0.032 | * |
|  |  |  |  |  |  |  |  |  | (0.015) |  |
| Married |  |  |  |  |  |  |  |  | 0.050 | ** |
|  |  |  |  |  |  |  |  |  | (0.018) |  |
| Pregnant |  |  |  |  |  |  |  |  | 0.076 | ** |
|  |  |  |  |  |  |  |  |  | (0.013) |  |
| Childbirth |  |  |  |  |  |  |  |  | -0.019 |  |
|  |  |  |  |  |  |  |  |  | (0.016) |  |
| Separated |  |  |  |  |  |  |  |  | -0.131 | ** |
|  |  |  |  |  |  |  |  |  | (0.018) |  |
| Reconciled |  |  |  |  |  |  |  |  | -0.062 |  |
|  |  |  |  |  |  |  |  |  | (0.034) |  |
| Moved |  |  |  |  |  |  |  |  | -0.001 |  |
|  |  |  |  |  |  |  |  |  | (0.007) |  |
| Spouse/child died |  |  |  |  |  |  |  |  | -0.157 | ** |
|  |  |  |  |  |  |  |  |  | (0.032) |  |
| Friend died |  |  |  |  |  |  |  |  | -0.009 |  |
|  |  |  |  |  |  |  |  |  | (0.008) |  |
| Relative died |  |  |  |  |  |  |  |  | -0.027 | ** |
|  |  |  |  |  |  |  |  |  | (0.007) |  |
| Health shock |  |  |  |  |  |  |  |  | -0.122 | ** |
|  |  |  |  |  |  |  |  |  | (0.010) |  |
| Physically attacked |  |  |  |  |  |  |  |  | -0.205 | ** |
|  |  |  |  |  |  |  |  |  | (0.032) |  |
| Family harmed |  |  |  |  |  |  |  |  | -0.023 | ** |
|  |  |  |  |  |  |  |  |  | (0.007) |  |
| Jailed |  |  |  |  |  |  |  |  | -0.170 | * |
|  |  |  |  |  |  |  |  |  | (0.077) |  |
| Relative jailed |  |  |  |  |  |  |  |  | -0.009 |  |
|  |  |  |  |  |  |  |  |  | (0.025) |  |
| Home lost |  |  |  |  |  |  |  |  | -0.001 |  |
|  |  |  |  |  |  |  |  |  | (0.020) |  |
| Intercept | 4.543 | ** | 4.457 | ** | 4.373 | ** | 4.498 | ** | 4.435 | ** |
|  | (0.016) |  | (0.033) |  | (0.061) |  | (0.060) |  | (0.066) |  |
| No. of observations | 115,630 |  | 115577 |  | 113951 |  | 113474 |  | 100873 |  |
| No. of individuals | 25716 |  | 25713 |  | 25628 |  | 25557 |  | 24360 |  |
| *Notes:* Figures are estimated coefficients from individual-level fixed-effects (within-person) models of hope. Standard errors clustered at the individual level are presented in parentheses. The estimates are based on the HILDA Survey data for years 2007, 2009, 2011, 2013, 2015, 2017, 2019, and 2021. The dependent hope variable ranges from 1 (totally hopeless) to 5 (totally hopeful). All included covariates are summarized in Table 3 and Supplementary Tables A1 and A2. Included in all regressions but not shown are survey year dummies. * and ** denote statistical significance at the 5% and 1% levels, respectively. | | | | | | | | | | |

Table A5. Determinants of Hope from Cross-Sectional and Panel Models by Gender, HILDA Survey

|  | | Pooled OLS models | | | | | | Fixed-effects models | | | | |  |  |
| --- | --- | --- | --- | --- | --- | --- | --- | --- | --- | --- | --- | --- | --- | --- |
|  | | Females | | | Males | | | Females | | | Males | |  |  |
| **Age group** | |  | |  |  | |  |  | |  |  | |  | |
| 25-34 | | 0.094 | | ** | 0.020 | |  | -0.013 | |  | -0.037 | |  | |
|  | | (0.017) | |  | (0.017) | |  | (0.021) | |  | (0.021) | |  | |
| 35-44 | | 0.180 | | ** | 0.096 | | ** | -0.030 | |  | -0.054 | |  | |
|  | | (0.020) | |  | (0.020) | |  | (0.029) | |  | (0.030) | |  | |
| 45-54 | | 0.273 | | ** | 0.207 | | ** | -0.011 | |  | -0.025 | |  | |
|  | | (0.019) | |  | (0.020) | |  | (0.036) | |  | (0.036) | |  | |
| 55-64 | | 0.373 | | ** | 0.289 | | ** | 0.034 | |  | 0.033 | |  | |
|  | | (0.021) | |  | (0.021) | |  | (0.044) | |  | (0.043) | |  | |
| 65-74 | | 0.507 | | ** | 0.353 | | ** | 0.108 | | * | 0.096 | |  | |
|  | | (0.024) | |  | (0.024) | |  | (0.051) | |  | (0.049) | |  | |
| 75-84 | | 0.581 | | ** | 0.403 | | ** | 0.159 | | ** | 0.129 | | * | |
|  | | (0.028) | |  | (0.028) | |  | (0.059) | |  | (0.058) | |  | |
| 85+ | | 0.627 | | ** | 0.362 | | ** | 0.209 | | ** | 0.081 | |  | |
|  | | (0.039) | |  | (0.038) | |  | (0.072) | |  | (0.073) | |  | |
| **Marital status** | |  | |  |  | |  |  | |  |  | |  | |
| Married | | 0.065 | | ** | -0.009 | |  | 0.042 | |  | 0.012 | |  | |
|  | | (0.017) | |  | (0.017) | |  | (0.026) | |  | (0.025) | |  | |
| De-facto | | 0.011 | |  | -0.023 | |  | 0.028 | |  | 0.003 | |  | |
|  | | (0.016) | |  | (0.016) | |  | (0.021) | |  | (0.020) | |  | |
| Separated | | 0.007 | |  | 0.038 | |  | -0.030 | |  | 0.046 | |  | |
|  | | (0.031) | |  | (0.034) | |  | (0.039) | |  | (0.040) | |  | |
| Divorced | | 0.052 | | * | 0.068 | | * | 0.050 | |  | 0.131 | | ** | |
|  | | (0.024) | |  | (0.029) | |  | (0.038) | |  | (0.041) | |  | |
| Widowed | | 0.080 | | ** | 0.190 | | ** | 0.030 | |  | 0.050 | |  | |
|  | | (0.026) | |  | (0.036) | |  | (0.045) | |  | (0.056) | |  | |
| **Household composition** | |  | |  |  | |  |  | |  |  | |  | |
| No. of children | | 0.017 | | ** | 0.006 | |  | 0.009 | |  | 0.009 | |  | |
|  | | (0.006) | |  | (0.005) | |  | (0.007) | |  | (0.006) | |  | |
| No. of adults | | -0.017 | | ** | -0.011 | | * | 0.001 | |  | 0.002 | |  | |
|  | | (0.005) | |  | (0.005) | |  | (0.006) | |  | (0.006) | |  | |
| **Education level** | |  | |  |  | |  |  | |  |  | |  | |
| University degree | | 0.047 | | ** | -0.018 | |  | -0.029 | |  | -0.022 | |  | |
|  | | (0.013) | |  | (0.015) | |  | (0.036) | |  | (0.034) | |  | |
| Diploma/certificate | | 0.042 | | ** | 0.006 | |  | 0.022 | |  | -0.038 | |  | |
|  | | (0.013) | |  | (0.013) | |  | (0.029) | |  | (0.030) | |  | |
| High school graduate | | 0.032 | | * | -0.022 | |  | -0.004 | |  | -0.062 | | * | |
|  | | (0.015) | |  | (0.015) | |  | (0.026) | |  | (0.024) | |  | |
| **Employment status** | |  | |  |  | |  |  | |  |  | |  | |
| Employed full-time | | 0.097 | | ** | 0.196 | | ** | 0.031 | | * | 0.104 | | ** | |
|  | | (0.014) | |  | (0.019) | |  | (0.015) | |  | (0.019) | |  | |
| Employed part-time | | 0.091 | | ** | 0.121 | | ** | 0.031 | | * | 0.063 | | ** | |
|  | | (0.013) | |  | (0.018) | |  | (0.013) | |  | (0.018) | |  | |
| Unemployed | | -0.071 | | ** | 0.010 | |  | -0.048 | |  | 0.012 | |  | |
|  | | (0.025) | |  | (0.026) | |  | (0.026) | |  | (0.027) | |  | |
| Retired | | 0.053 | | ** | 0.135 | | ** | 0.020 | |  | 0.097 | | ** | |
|  | | (0.015) | |  | (0.022) | |  | (0.015) | |  | (0.020) | |  | |
| **Household income** | |  | |  |  | |  |  | |  |  | |  | |
| Log of HH income | | 0.011 | |  | 0.027 | | ** | 0.002 | |  | 0.018 | | * | |
|  | | (0.007) | |  | (0.007) | |  | (0.008) | |  | (0.008) | |  | |
| Neighborhood SES | | 0.001 | |  | -0.000 | |  | 0.002 | |  | 0.007 | | ** | |
|  | | (0.002) | |  | (0.002) | |  | (0.003) | |  | (0.003) | |  | |
| **Living status** | |  | |  |  | |  |  | |  |  | |  | |
| Homeowner | | 0.021 | | * | 0.043 | | ** | -0.015 | |  | 0.016 | |  | |
|  | | (0.011) | |  | (0.011) | |  | (0.013) | |  | (0.013) | |  | |
| Lives in major city | | -0.046 | | ** | -0.042 | | ** | -0.064 | | * | -0.045 | |  | |
|  | | (0.015) | |  | (0.015) | |  | (0.029) | |  | (0.028) | |  | |
| Lives in inner regional | | -0.028 | |  | -0.013 | |  | -0.045 | |  | -0.015 | |  | |
|  | | (0.015) | |  | (0.015) | |  | (0.029) | |  | (0.028) | |  | |
| Migrant | | -0.030 | | ** | -0.024 | | * |  | |  |  | |  | |
|  | | (0.012) | |  | (0.012) | |  |  | |  |  | |  | |
| **Health status** | |  | |  |  | |  |  | |  |  | |  | |
| Poor health | | -0.370 | | ** | -0.300 | | ** | -0.228 | | ** | -0.201 | | ** | |
|  | | (0.014) | |  | (0.015) | |  | (0.014) | |  | (0.015) | |  | |
| Long-term health condition | | -0.047 | | ** | -0.057 | | ** | -0.027 | | ** | -0.027 | | ** | |
|  | | (0.010) | |  | (0.010) | |  | (0.010) | |  | (0.010) | |  | |
| Smoke every day | | -0.084 | | ** | -0.036 | | ** | -0.038 | |  | -0.008 | |  | |
|  | | (0.015) | |  | (0.014) | |  | (0.020) | |  | (0.019) | |  | |
| Drink alcohol every day | | -0.048 | | ** | -0.030 | | ** | -0.063 | | ** | -0.042 | | ** | |
|  | | (0.012) | |  | (0.010) | |  | (0.015) | |  | (0.012) | |  | |
| BMI: Underweight | | 0.012 | |  | 0.023 | |  | -0.019 | |  | -0.032 | |  | |
|  | | (0.025) | |  | (0.032) | |  | (0.030) | |  | (0.043) | |  | |
| BMI: Healthy Weight | | 0.027 | | * | 0.027 | | * | 0.016 | |  | -0.001 | |  | |
|  | | (0.011) | |  | (0.011) | |  | (0.015) | |  | (0.016) | |  | |
| BMI: Overweight | | 0.012 | |  | 0.010 | |  | 0.018 | |  | 0.006 | |  | |
|  | | (0.011) | |  | (0.010) | |  | (0.013) | |  | (0.012) | |  | |
| **Social life** | |  | |  |  | |  |  | |  |  | |  | |
| Meet with friends  almost every day | | 0.016 | | * | 0.019 | | * | 0.040 | | ** | 0.017 | |  | |
|  | | (0.008) | |  | (0.009) | |  | (0.008) | |  | (0.009) | |  | |
| Have lots of friends | | 0.031 | | ** | 0.030 | | ** | 0.039 | | ** | 0.037 | | ** | |
|  | | (0.003) | |  | (0.003) | |  | (0.003) | |  | (0.003) | |  | |
| Don’t get much help | | -0.063 | | ** | -0.059 | | ** | -0.042 | | ** | -0.042 | | ** | |
|  | | (0.003) | |  | (0.003) | |  | (0.003) | |  | (0.003) | |  | |
| No one to lean on | | -0.032 | | ** | -0.023 | | ** | -0.025 | | ** | -0.020 | | ** | |
|  | | (0.003) | |  | (0.003) | |  | (0.003) | |  | (0.003) | |  | |
| Feel very lonely | | -0.130 | | ** | -0.135 | | ** | -0.088 | | ** | -0.085 | | ** | |
|  | | (0.003) | |  | (0.004) | |  | (0.003) | |  | (0.003) | |  | |
| **Major life events** | |  | |  |  | |  |  | |  |  | |  | |
| Money gained | | 0.048 | | ** | -0.006 | |  | 0.019 | |  | -0.009 | |  | |
|  | | (0.018) | |  | (0.018) | |  | (0.017) | |  | (0.017) | |  | |
| Money lost | | -0.288 | | ** | -0.275 | | ** | -0.243 | | ** | -0.231 | | ** | |
|  | | (0.028) | |  | (0.029) | |  | (0.026) | |  | (0.028) | |  | |
| Hired | | 0.009 | |  | 0.020 | |  | 0.022 | | * | 0.026 | | * | |
|  | | (0.011) | |  | (0.010) | |  | (0.011) | |  | (0.011) | |  | |
| Fired | | -0.082 | | ** | -0.052 | | ** | -0.070 | | ** | -0.025 | |  | |
|  | | (0.026) | |  | (0.020) | |  | (0.024) | |  | (0.019) | |  | |
| Promoted | | 0.037 | | ** | 0.033 | | ** | 0.041 | | ** | 0.042 | | ** | |
|  | | (0.014) | |  | (0.012) | |  | (0.014) | |  | (0.013) | |  | |
| Retired | | 0.026 | |  | -0.028 | |  | -0.013 | |  | -0.048 | | * | |
|  | | (0.022) | |  | (0.024) | |  | (0.022) | |  | (0.021) | |  | |
| Married | | -0.026 | |  | -0.039 | |  | 0.027 | |  | 0.002 | |  | |
|  | | (0.025) | |  | (0.022) | |  | (0.024) | |  | (0.023) | |  | |
| Pregnant | | 0.084 | | ** | 0.012 | |  | 0.065 | | ** | 0.031 | |  | |
|  | | (0.018) | |  | (0.017) | |  | (0.018) | |  | (0.018) | |  | |
| Childbirth | | 0.016 | |  | -0.003 | |  | -0.008 | |  | -0.018 | |  | |
|  | | (0.023) | |  | (0.020) | |  | (0.023) | |  | (0.021) | |  | |
| Separated | | -0.117 | | ** | -0.130 | | ** | -0.070 | | ** | -0.123 | | ** | |
|  | | (0.023) | |  | (0.024) | |  | (0.023) | |  | (0.025) | |  | |
| Reconciled | | -0.083 | |  | -0.066 | |  | -0.063 | |  | -0.038 | |  | |
|  | | (0.043) | |  | (0.049) | |  | (0.043) | |  | (0.049) | |  | |
| Moved | | -0.005 | |  | -0.012 | |  | -0.003 | |  | -0.005 | |  | |
|  | | (0.010) | |  | (0.010) | |  | (0.010) | |  | (0.010) | |  | |
| Spouse/child died | | -0.185 | | ** | -0.171 | | ** | -0.168 | | ** | -0.122 | | * | |
|  | | (0.045) | |  | (0.053) | |  | (0.041) | |  | (0.055) | |  | |
| Friend died | | -0.042 | | ** | -0.036 | | ** | -0.012 | |  | -0.012 | |  | |
|  | | (0.012) | |  | (0.011) | |  | (0.011) | |  | (0.011) | |  | |
| Relative died | | -0.025 | | * | -0.020 | |  | -0.021 | | * | -0.023 | | * | |
|  | | (0.010) | |  | (0.011) | |  | (0.010) | |  | (0.010) | |  | |
| Health shock | | -0.100 | | ** | -0.068 | | ** | -0.074 | | ** | -0.084 | | ** | |
|  | | (0.015) | |  | (0.014) | |  | (0.013) | |  | (0.013) | |  | |
| Physically attacked | | -0.244 | | ** | -0.242 | | ** | -0.163 | | ** | -0.167 | | ** | |
|  | | (0.042) | |  | (0.040) | |  | (0.040) | |  | (0.045) | |  | |
| Family harmed | | -0.018 | |  | -0.010 | |  | -0.025 | | ** | -0.005 | |  | |
|  | | (0.009) | |  | (0.010) | |  | (0.009) | |  | (0.009) | |  | |
| Jailed | | -0.094 | |  | -0.262 | | ** | 0.023 | |  | -0.284 | | ** | |
|  | | (0.135) | |  | (0.074) | |  | (0.155) | |  | (0.085) | |  | |
| Relative jailed | | 0.021 | |  | -0.021 | |  | 0.010 | |  | -0.033 | |  | |
|  | | (0.030) | |  | (0.034) | |  | (0.032) | |  | (0.037) | |  | |
| Home lost | | -0.023 | |  | -0.056 | |  | 0.010 | |  | -0.004 | |  | |
|  | | (0.031) | |  | (0.029) | |  | (0.026) | |  | (0.028) | |  | |
| Intercept | | 4.632 | | ** | 4.560 | | ** | 4.732 | | ** | 4.583 | | ** | |
|  | | (0.075) | |  | (0.081) | |  | (0.093) | |  | (0.096) | |  | |
| No. of observations | | 52401 | |  | 46334 | |  | 52401 | |  | 46334 | |  | |
| No. of individuals | |  | |  |  | |  | 12613 | |  | 11598 | |  | |
| *Notes:* Figures are estimated coefficients from cross-sectional (pooled) OLS and fixed-effects (within-person) models of hope for male and female subsamples. Standard errors clustered at the individual level are presented in parentheses. The estimates are based on the HILDA Survey data for years 2007, 2009, 2011, 2013, 2015, 2017, 2019, and 2021. The dependent hope variable ranges from 1 (totally hopeless) to 5 (totally hopeful). All included covariates are summarized in Table 3 and Supplementary Tables A1 and A2. Included in all regressions but not shown are survey year dummies. * and ** denote statistical significance at the 5% and 1% levels, respectively. | | | | | | | | | | | | | | |

Table A6. Determinants of Hope from Cross-Sectional and Panel Models by Income, HILDA Survey

|  | Pooled OLS models | | | | Fixed-effects models | | | |
| --- | --- | --- | --- | --- | --- | --- | --- | --- |
|  | Poor | | Rich | | Poor | | Rich | |
| Male | 0.072 | ** | 0.087 | ** |  |  |  |  |
|  | (0.015) |  | (0.010) |  |  |  |  |  |
| **Age group** |  |  |  |  |  |  |  |  |
| 25-34 | -0.008 |  | 0.094 | ** | -0.143 | ** | 0.017 |  |
|  | (0.027) |  | (0.024) |  | (0.049) |  | (0.034) |  |
| 35-44 | 0.112 | ** | 0.171 | ** | -0.153 | * | -0.032 |  |
|  | (0.035) |  | (0.026) |  | (0.076) |  | (0.044) |  |
| 45-54 | 0.257 | ** | 0.249 | ** | -0.110 |  | -0.028 |  |
|  | (0.034) |  | (0.025) |  | (0.094) |  | (0.051) |  |
| 55-64 | 0.377 | ** | 0.298 | ** | -0.077 |  | -0.003 |  |
|  | (0.034) |  | (0.027) |  | (0.106) |  | (0.060) |  |
| 65-74 | 0.497 | ** | 0.369 | ** | -0.006 |  | 0.012 |  |
|  | (0.036) |  | (0.031) |  | (0.114) |  | (0.070) |  |
| 75-84 | 0.547 | ** | 0.437 | ** | -0.000 |  | 0.032 |  |
|  | (0.038) |  | (0.039) |  | (0.123) |  | (0.086) |  |
| 85+ | 0.559 | ** | 0.279 | * | -0.022 |  | -0.065 |  |
|  | (0.044) |  | (0.109) |  | (0.134) |  | (0.162) |  |
| **Marital status** |  |  |  |  |  |  |  |  |
| Married | 0.013 |  | 0.060 | ** | 0.063 |  | 0.050 |  |
|  | (0.028) |  | (0.023) |  | (0.082) |  | (0.041) |  |
| De-facto | 0.009 |  | 0.004 |  | 0.045 |  | 0.017 |  |
|  | (0.028) |  | (0.023) |  | (0.054) |  | (0.036) |  |
| Separated | -0.001 |  | 0.063 |  | 0.072 |  | 0.072 |  |
|  | (0.037) |  | (0.051) |  | (0.089) |  | (0.068) |  |
| Divorced | 0.037 |  | 0.130 | ** | 0.098 |  | 0.180 | * |
|  | (0.029) |  | (0.047) |  | (0.085) |  | (0.071) |  |
| Widowed | 0.079 | ** | 0.110 | * | 0.034 |  | 0.162 |  |
|  | (0.031) |  | (0.054) |  | (0.085) |  | (0.108) |  |
| **Household composition** |  |  |  |  |  |  |  |  |
| No. of children | 0.045 | ** | 0.002 |  | 0.023 |  | 0.029 | ** |
|  | (0.014) |  | (0.005) |  | (0.026) |  | (0.009) |  |
| No. of adults | -0.031 | * | -0.009 |  | -0.022 |  | 0.006 |  |
|  | (0.014) |  | (0.005) |  | (0.021) |  | (0.007) |  |
| **Education level** |  |  |  |  |  |  |  |  |
| University degree | 0.038 |  | -0.002 |  | -0.136 |  | -0.066 |  |
|  | (0.020) |  | (0.016) |  | (0.092) |  | (0.046) |  |
| Diploma/certificate | 0.013 |  | 0.024 |  | -0.061 |  | -0.022 |  |
|  | (0.017) |  | (0.016) |  | (0.071) |  | (0.045) |  |
| High school graduate | -0.002 |  | 0.020 |  | -0.135 |  | -0.019 |  |
|  | (0.023) |  | (0.017) |  | (0.080) |  | (0.032) |  |
| **Employment status** |  |  |  |  |  |  |  |  |
| Employed full-time | 0.197 | ** | 0.064 | ** | 0.091 | ** | 0.012 |  |
|  | (0.024) |  | (0.019) |  | (0.034) |  | (0.025) |  |
| Employed part-time | 0.124 | ** | 0.053 | ** | 0.083 | ** | 0.003 |  |
|  | (0.021) |  | (0.019) |  | (0.027) |  | (0.023) |  |
| Unemployed | -0.032 |  | -0.065 |  | -0.015 |  | -0.047 |  |
|  | (0.033) |  | (0.036) |  | (0.041) |  | (0.043) |  |
| Retired | 0.086 | ** | 0.016 |  | 0.043 | * | 0.001 |  |
|  | (0.020) |  | (0.026) |  | (0.021) |  | (0.031) |  |
| **Living status** |  |  |  |  |  |  |  |  |
| Homeowner | 0.054 | ** | -0.003 |  | -0.032 |  | -0.011 |  |
|  | (0.017) |  | (0.014) |  | (0.029) |  | (0.018) |  |
| Lives in major city | 0.354 |  | 0.316 |  | 0.033 |  | 0.361 |  |
|  | (0.419) |  | (0.386) |  | (0.384) |  | (0.440) |  |
| Lives in inner regional | 0.401 |  | 0.319 |  | 0.109 |  | 0.352 |  |
|  | (0.419) |  | (0.386) |  | (0.387) |  | (0.441) |  |
| Lives in rural | 0.414 |  | 0.356 |  | 0.041 |  | 0.392 |  |
|  | (0.419) |  | (0.387) |  | (0.386) |  | (0.444) |  |
| Migrant | -0.022 |  | -0.008 |  |  |  |  |  |
|  | (0.017) |  | (0.012) |  |  |  |  |  |
| **Health status** |  |  |  |  |  |  |  |  |
| Poor health | -0.346 | ** | -0.272 | ** | -0.190 | ** | -0.168 | ** |
|  | (0.017) |  | (0.020) |  | (0.019) |  | (0.024) |  |
| Long-term health condition | -0.067 | ** | -0.037 | ** | -0.038 | ** | -0.024 |  |
|  | (0.013) |  | (0.014) |  | (0.014) |  | (0.016) |  |
| Smoke every day | -0.093 | ** | -0.069 | ** | -0.015 |  | -0.026 |  |
|  | (0.020) |  | (0.019) |  | (0.034) |  | (0.035) |  |
| Drink alcohol every day | -0.027 |  | -0.029 | * | -0.053 | * | -0.053 | ** |
|  | (0.016) |  | (0.012) |  | (0.025) |  | (0.017) |  |
| BMI: Underweight | -0.017 |  | 0.035 |  | -0.084 |  | -0.057 |  |
|  | (0.039) |  | (0.033) |  | (0.058) |  | (0.049) |  |
| BMI: Healthy Weight | 0.058 | ** | 0.022 |  | 0.025 |  | -0.020 |  |
|  | (0.016) |  | (0.013) |  | (0.025) |  | (0.022) |  |
| BMI: Overweight | 0.035 | * | -0.000 |  | 0.008 |  | -0.012 |  |
|  | (0.016) |  | (0.012) |  | (0.021) |  | (0.018) |  |
| **Social life** |  |  |  |  |  |  |  |  |
| Meet with friends  almost every day | 0.028 | * | 0.011 |  | 0.045 | ** | 0.037 | ** |
|  | (0.012) |  | (0.010) |  | (0.014) |  | (0.013) |  |
| Have lots of friends | 0.043 | ** | 0.023 | ** | 0.042 | ** | 0.033 | ** |
|  | (0.004) |  | (0.003) |  | (0.005) |  | (0.005) |  |
| Don’t get much help | -0.062 | ** | -0.063 | ** | -0.036 | ** | -0.045 | ** |
|  | (0.004) |  | (0.004) |  | (0.005) |  | (0.005) |  |
| No one to lean on | -0.035 | ** | -0.021 | ** | -0.015 | ** | -0.021 | ** |
|  | (0.004) |  | (0.004) |  | (0.004) |  | (0.005) |  |
| Feel very lonely | -0.103 | ** | -0.145 | ** | -0.050 | ** | -0.099 | ** |
|  | (0.004) |  | (0.004) |  | (0.004) |  | (0.005) |  |
| **Major life events** |  |  |  |  |  |  |  |  |
| Money gained | 0.061 |  | 0.014 |  | 0.044 |  | 0.010 |  |
|  | (0.037) |  | (0.017) |  | (0.038) |  | (0.019) |  |
| Money lost | -0.299 | ** | -0.294 | ** | -0.231 | ** | -0.199 | ** |
|  | (0.036) |  | (0.050) |  | (0.039) |  | (0.056) |  |
| Hired | 0.034 |  | -0.015 |  | 0.044 |  | -0.002 |  |
|  | (0.021) |  | (0.012) |  | (0.027) |  | (0.014) |  |
| Fired | -0.087 | * | -0.018 |  | -0.078 |  | 0.029 |  |
|  | (0.041) |  | (0.025) |  | (0.047) |  | (0.028) |  |
| Promoted | 0.016 |  | 0.036 | ** | 0.077 |  | 0.043 | ** |
|  | (0.035) |  | (0.014) |  | (0.048) |  | (0.015) |  |
| Retired | -0.030 |  | -0.003 |  | -0.048 |  | -0.016 |  |
|  | (0.030) |  | (0.032) |  | (0.030) |  | (0.034) |  |
| Married | -0.122 |  | -0.039 |  | -0.137 |  | 0.046 |  |
|  | (0.068) |  | (0.028) |  | (0.098) |  | (0.032) |  |
| Pregnant | 0.070 |  | 0.024 |  | 0.127 |  | 0.024 |  |
|  | (0.048) |  | (0.020) |  | (0.068) |  | (0.023) |  |
| Childbirth | -0.012 |  | 0.031 |  | -0.159 |  | 0.014 |  |
|  | (0.061) |  | (0.025) |  | (0.082) |  | (0.028) |  |
| Separated | -0.122 | ** | -0.093 | ** | -0.079 | * | -0.110 | * |
|  | (0.030) |  | (0.036) |  | (0.037) |  | (0.045) |  |
| Reconciled | -0.104 |  | -0.125 |  | -0.068 |  | -0.087 |  |
|  | (0.065) |  | (0.082) |  | (0.076) |  | (0.099) |  |
| Moved | -0.012 |  | -0.004 |  | 0.018 |  | -0.019 |  |
|  | (0.017) |  | (0.013) |  | (0.020) |  | (0.014) |  |
| Spouse/child died | -0.156 | ** | -0.145 |  | -0.166 | ** | -0.227 | * |
|  | (0.049) |  | (0.086) |  | (0.046) |  | (0.111) |  |
| Friend died | -0.024 |  | -0.049 | ** | -0.012 |  | -0.033 |  |
|  | (0.015) |  | (0.016) |  | (0.015) |  | (0.017) |  |
| Relative died | -0.035 |  | -0.010 |  | -0.006 |  | 0.005 |  |
|  | (0.018) |  | (0.013) |  | (0.018) |  | (0.013) |  |
| Health shock | -0.101 | ** | -0.065 | ** | -0.114 | ** | -0.064 | ** |
|  | (0.020) |  | (0.019) |  | (0.020) |  | (0.019) |  |
| Physically attacked | -0.197 | ** | -0.255 | ** | -0.108 |  | -0.087 |  |
|  | (0.052) |  | (0.061) |  | (0.062) |  | (0.077) |  |
| Family harmed | 0.005 |  | -0.014 |  | 0.002 |  | -0.034 | ** |
|  | (0.016) |  | (0.012) |  | (0.016) |  | (0.012) |  |
| Jailed | -0.179 |  | -0.235 |  | -0.140 |  | -0.123 |  |
|  | (0.094) |  | (0.190) |  | (0.127) |  | (0.132) |  |
| Relative jailed | 0.042 |  | -0.037 |  | -0.023 |  | -0.051 |  |
|  | (0.043) |  | (0.049) |  | (0.053) |  | (0.061) |  |
| Home lost | -0.084 |  | -0.012 |  | -0.012 |  | 0.044 |  |
|  | (0.050) |  | (0.039) |  | (0.049) |  | (0.043) |  |
| Intercept | 4.202 | ** | 4.541 | ** | 4.663 | ** | 4.513 | ** |
|  | (0.422) |  | (0.389) |  | (0.403) |  | (0.447) |  |
| No. of observations | 22816 |  | 26699 |  | 22816 |  | 26699 |  |
| No. of individuals |  |  |  |  | 9221 |  | 11235 |  |
| *Notes:* Figures are estimated coefficients from cross-sectional (pooled) OLS and fixed-effects (within-person) models of hope for poor and rich subsamples. Poor subsample captures individuals in the bottom income quartile. Rich subsample captures individuals in the top income quartile. Standard errors clustered at the individual level are presented in parentheses. The estimates are based on the HILDA Survey data for years 2007, 2009, 2011, 2013, 2015, 2017, 2019, and 2021. The dependent hope variable ranges from 1 (totally hopeless) to 5 (totally hopeful). All included covariates are summarized in Table 3 and Supplementary Tables A1 and A2. Included in all regressions but not shown are survey year dummies. * and ** denote statistical significance at the 5% and 1% levels, respectively. | | | | | | | | |

Table A7. Leads and Lags in Hope to Major Life Events, HILDA Survey

| *Life event:* | Money  gained | Money  lost | Promoted | Fired | Married | Separated |
| --- | --- | --- | --- | --- | --- | --- |
| *Occurs in 4-5 years* | 0.011 | -0.117 | 0.020 | 0.048 | -0.017 | -0.041 |
|  | (0.028) | (0.056) | (0.027) | (0.043) | (0.060) | (0.041) |
| *Occurs in 2-3 years* | 0.038 | -0.159** | 0.009 | 0.010 | 0.056 | 0.003 |
|  | (0.033) | (0.061) | (0.030) | (0.044) | (0.057) | (0.051) |
| Occurred 0-1 years ago | 0.003 | -0.382** | 0.048 | -0.007 | 0.037 | -0.124* |
|  | (0.036) | (0.064) | (0.031) | (0.050) | (0.059) | (0.051) |
| *Occurred 2-3 years ago* | -0.025 | -0.032 | 0.029 | -0.002 | 0.050 | -0.026 |
|  | (0.037) | (0.050) | (0.029) | (0.046) | (0.048) | (0.045) |
| *Occurred 4-5 years ago* | 0.014 | 0.012 | -0.014 | -0.005 | -0.017 | -0.032 |
|  | (0.031) | (0.036) | (0.023) | (0.037) | (0.060) | (0.038) |

*Notes:* Figures are estimated coefficients from one fixed-effects (within-person) model of hope. Standard errors clustered at the individual level are presented in parentheses. The dependent hope variable ranges from 1 (totally hopeless) to 5 (totally hopeful). All reported life events are summarised in Table 3. Included in the hope equation but not shown are the covariates age, employment status, household income, and year dummies. Number of individuals and person-year observations equals 10,284 and 24,089 respectively. * and ** denote statistical significance at the 5% and 1% levels respectively.

Table A7 *continued.*

| *Life event:* | Pregnancy | Birth | Death of spouse/child | Illness | Physically attacked | Jailed | Lost home |
| --- | --- | --- | --- | --- | --- | --- | --- |
| *Occurs in 4-5 years* | 0.042 | 0.017 | -0.048 | -0.022 | -0.137 | 0.082 | -0.025 |
|  | (0.041) | (0.046) | (0.060) | (0.021) | (0.082) | (0.279) | (0.063) |
| *Occurs in 2-3 years* | 0.036 | 0.014 | 0.008 | -0.040 | -0.187 | -0.162 | -0.072 |
|  | (0.045) | (0.051) | (0.075) | (0.023) | (0.099) | (0.220) | (0.062) |
| Occurred 0-1 years ago | 0.095* | 0.056 | -0.240* | -0.151** | -0.236* | -0.500 | -0.079 |
|  | (0.041) | (0.050) | (0.099) | (0.026) | (0.098) | (0.289) | (0.056) |
| *Occurred 2-3 years ago* | 0.027 | 0.068 | -0.058 | -0.065** | 0.039 | -0.424 | -0.019 |
|  | (0.036) | (0.044) | (0.088) | (0.024) | (0.084) | (0.226) | (0.050) |
| *Occurred 4-5 years ago* | 0.001 | -0.012 | 0.074 | -0.069** | 0.045 | -0.098 | 0.011 |
|  | (0.032) | (0.037) | (0.078) | (0.022) | (0.071) | (0.208) | (0.044) |

Table A8. Leads and Lags in Hope to Major Life Events by Gender, HILDA Survey

| *Life event:* | Money gained | | Money lost | | Promoted | | Fired | |
| --- | --- | --- | --- | --- | --- | --- | --- | --- |
|  | Females | Males | Females | Males | Females | Males | Females | Males |
| *Occurs in 4-5 years* | 0.038 | -0.025 | -0.106 | -0.122 | 0.013 | 0.017 | 0.020 | 0.066 |
|  | (0.038) | (0.043) | (0.072) | (0.087) | (0.040) | (0.034) | (0.070) | (0.053) |
| *Occurs in 2-3 years* | 0.060 | 0.015 | -0.141 | -0.180* | 0.017 | 0.002 | 0.012 | 0.006 |
|  | (0.046) | (0.048) | (0.080) | (0.091) | (0.045) | (0.039) | (0.070) | (0.057) |
| Occurred 0-1 years ago | 0.027 | -0.021 | -0.302** | -0.489** | 0.052 | 0.050 | -0.059 | 0.041 |
|  | (0.050) | (0.052) | (0.085) | (0.096) | (0.046) | (0.040) | (0.083) | (0.061) |
| *Occurred 2-3 years ago* | -0.031 | -0.019 | -0.009 | -0.052 | 0.049 | 0.015 | 0.007 | -0.011 |
|  | (0.050) | (0.054) | (0.068) | (0.070) | (0.043) | (0.038) | (0.076) | (0.056) |
| *Occurred 4-5 years ago* | 0.031 | -0.001 | 0.064 | -0.046 | 0.001 | -0.029 | -0.026 | 0.008 |
|  | (0.043) | (0.045) | (0.053) | (0.049) | (0.033) | (0.031) | (0.060) | (0.045) |

*Notes:* Figures are estimated coefficients from two fixed-effects (within-person) models of hope for males and females. Standard errors clustered at the individual level are presented in parentheses. The dependent hope variable ranges from 1 (totally hopeless) to 5 (totally hopeful). All reported life events are summarised in Table 3. Included in the hope equations but not shown are the covariates age, employment status, household income, and year dummies. Number of person-year observations equals 13,126 for females and 10,963 for males. * and ** denote statistical significance at the 5% and 1% levels respectively.

Table A8 *continued.*

| *Life event:* | Married | | Separated | | Pregnancy | | Birth | |
| --- | --- | --- | --- | --- | --- | --- | --- | --- |
|  | Females | Males | Females | Males | Females | Males | Females | Males |
| *Occurs in 4-5 years* | -0.093 | 0.065 | -0.062 | -0.003 | 0.010 | 0.082 | 0.044 | -0.026 |
|  | (0.091) | (0.072) | (0.055) | (0.059) | (0.056) | (0.061) | (0.066) | (0.063) |
| *Occurs in 2-3 years* | 0.123 | -0.035 | 0.039 | -0.042 | -0.065 | 0.170** | 0.100 | -0.084 |
|  | (0.083) | (0.077) | (0.071) | (0.070) | (0.064) | (0.059) | (0.070) | (0.070) |
| Occurred 0-1 years ago | 0.112 | -0.063 | -0.083 | -0.172* | 0.092 | 0.094 | 0.087 | 0.001 |
|  | (0.083) | (0.080) | (0.068) | (0.074) | (0.059) | (0.054) | (0.071) | (0.066) |
| *Occurred 2-3 years ago* | 0.095 | -0.015 | 0.061 | -0.161** | 0.051 | 0.003 | 0.030 | 0.096 |
|  | (0.065) | (0.072) | (0.062) | (0.061) | (0.051) | (0.049) | (0.065) | (0.056) |
| *Occurred 4-5 years ago* | -0.064 | -0.007 | 0.009 | -0.092 | 0.031 | -0.043 | -0.026 | 0.001 |
|  | (0.059) | (0.049) | (0.053) | (0.052) | (0.045) | (0.045) | (0.054) | (0.049) |

Table A8 *continued.*

| *Life event:* | Death of spouse/child | | Illness | | Jailed | | Lost home | |
| --- | --- | --- | --- | --- | --- | --- | --- | --- |
|  | Females | Males | Females | Males | Females | Males | Females | Males |
| *Occurs in 4-5 years* | -0.122 | 0.107 | -0.029 | -0.013 | 0.607 | -0.094 | -0.043 | -0.018 |
|  | (0.076) | (0.085) | (0.031) | (0.028) | (0.412) | (0.314) | (0.087) | (0.090) |
| *Occurs in 2-3 years* | -0.024 | 0.070 | -0.043 | -0.027 | -0.092 | -0.101 | -0.080 | -0.052 |
|  | (0.083) | (0.148) | (0.034) | (0.032) | (0.327) | (0.275) | (0.092) | (0.083) |
| Occurred 0-1 years ago | -0.276* | -0.220 | -0.123** | -0.167** | -0.593 | -0.310 | -0.076 | -0.066 |
|  | (0.108) | (0.187) | (0.037) | (0.036) | (0.365) | (0.385) | (0.081) | (0.077) |
| *Occurred 2-3 years ago* | -0.118 | 0.026 | -0.035 | -0.092** | -0.243 | -0.386 | -0.107 | 0.084 |
|  | (0.089) | (0.184) | (0.032) | (0.034) | (0.446) | (0.247) | (0.073) | (0.065) |
| *Occurred 4-5 years ago* | 0.009 | 0.191 | -0.078* | -0.053 | 0.407 | -0.094 | -0.008 | 0.029 |
|  | (0.086) | (0.144) | (0.030) | (0.031) | (0.478) | (0.314) | (0.063) | (0.060) |

Table A8 *continued.*

| *Life event:* | Physically attacked | |
| --- | --- | --- |
|  | Females | Males |
| *Occurs in 4-5 years* | -0.226* | 0.029 |
|  | (0.108) | (0.119) |
| *Occurs in 2-3 years* | -0.271* | 0.033 |
|  | (0.133) | (0.144) |
| Occurred 0-1 years ago | -0.273* | -0.166 |
|  | (0.130) | (0.150) |
| *Occurred 2-3 years ago* | 0.082 | -0.029 |
|  | (0.115) | (0.121) |
| *Occurred 4-5 years ago* | 0.010 | 0.076 |
|  | (0.103) | (0.095) |

Table A9. Leads and Lags in Hope to Major Life Events by Income, HILDA Survey

| *Life event:* | Money gained | | Money lost | | Promoted | | Fired | |
| --- | --- | --- | --- | --- | --- | --- | --- | --- |
|  | Poor | Rich | Poor | Rich | Poor | Rich | Poor | Rich |
| *Occurs in 4-5 years* | 0.054 | 0.036 | -0.191 | -0.093 | 0.284* | 0.022 | -0.003 | -0.023 |
|  | (0.066) | (0.047) | (0.149) | (0.161) | (0.122) | (0.041) | (0.140) | (0.080) |
| *Occurs in 2-3 years* | 0.037 | 0.039 | -0.185 | -0.252 | 0.135 | 0.054 | -0.122 | 0.038 |
|  | (0.072) | (0.059) | (0.156) | (0.168) | (0.186) | (0.042) | (0.154) | (0.084) |
| Occurred 0-1 years ago | 0.116 | -0.018 | -0.372** | -0.365 | 0.325 | 0.009 | -0.190 | -0.015 |
|  | (0.109) | (0.062) | (0.139) | (0.198) | (0.168) | (0.044) | (0.134) | (0.102) |
| *Occurred 2-3 years ago* | -0.091 | -0.034 | -0.071 | 0.200 | -0.031 | -0.002 | -0.127 | 0.058 |
|  | (0.085) | (0.063) | (0.116) | (0.147) | (0.150) | (0.047) | (0.103) | (0.083) |
| *Occurred 4-5 years ago* | 0.020 | 0.036 | 0.012 | -0.020 | -0.028 | -0.021 | -0.111 | -0.023 |
|  | (0.057) | (0.047) | (0.081) | (0.102) | (0.133) | (0.037) | (0.101) | (0.077) |

*Notes:* Figures are estimated coefficients from two fixed-effects (within-person) models of hope for poor and rich subsamples. Poor subsample captures individuals in the bottom income quartile. Rich subsample captures individuals in the top income quartile. Standard errors clustered at the individual level are presented in parentheses. The dependent hope variable ranges from 1 (totally hopeless) to 5 (totally hopeful). All reported life events are summarised in Table 3. Included in the hope equations but not shown are the covariates age, employment status, household income, and year dummies. Number of person-year observations equals 5,585 for poor subsample and 6,448 for rich subsample. * and ** denote statistical significance at the 5% and 1% levels respectively.

Table A9 *continued.*

| *Life event:* | Married | | Separated | | Pregnancy | | Birth | |
| --- | --- | --- | --- | --- | --- | --- | --- | --- |
|  | Poor | Rich | Poor | Rich | Poor | Rich | Poor | Rich |
| *Occurs in 4-5 years* | -0.088 | 0.122 | -0.295* | -0.014 | 0.025 | -0.059 | 0.100 | 0.012 |
|  | (0.200) | (0.138) | (0.133) | (0.099) | (0.250) | (0.082) | (0.341) | (0.113) |
| *Occurs in 2-3 years* | 0.070 | 0.070 | 0.159 | -0.146 | -0.435 | -0.087 | -0.084 | -0.170 |
|  | (0.213) | (0.142) | (0.121) | (0.136) | (0.305) | (0.090) | (0.361) | (0.119) |
| Occurred 0-1 years ago | 0.455 | 0.111 | -0.207 | -0.420** | -0.167 | -0.047 | -0.137 | 0.004 |
|  | (0.385) | (0.128) | (0.114) | (0.161) | (0.322) | (0.071) | (0.308) | (0.093) |
| *Occurred 2-3 years ago* | 0.387 | 0.048 | -0.130 | -0.089 | -0.685** | 0.071 | 0.835** | -0.065 |
|  | (0.276) | (0.098) | (0.093) | (0.169) | (0.236) | (0.056) | (0.292) | (0.069) |
| *Occurred 4-5 years ago* | 0.240 | -0.048 | 0.035 | -0.177 | -0.147 | 0.047 | 0.069 | -0.121 |
|  | (0.148) | (0.102) | (0.091) | (0.113) | (0.179) | (0.055) | (0.255) | (0.062) |

Table A9 *continued.*

| *Life event:* | Death of spouse/child | | Illness | | Jailed | | Lost home | |
| --- | --- | --- | --- | --- | --- | --- | --- | --- |
|  | Poor | Rich | Poor | Rich | Poor | Rich | Poor | Rich |
| *Occurs in 4-5 years* | -0.063 | -0.086 | -0.005 | -0.031 | 0.143 | -1.243** | -0.037 | 0.194 |
|  | (0.099) | (0.169) | (0.041) | (0.046) | (0.423) | (0.471) | (0.129) | (0.135) |
| *Occurs in 2-3 years* | -0.046 | -0.085 | -0.025 | -0.036 | -0.082 | -0.345 | -0.287 | 0.207 |
|  | (0.111) | (0.133) | (0.047) | (0.051) | (0.300) | (0.830) | (0.167) | (0.122) |
| Occurred 0-1 years ago | -0.322* | -0.336 | -0.150** | -0.133* | -0.821 | 0.197 | -0.193 | 0.030 |
|  | (0.132) | (0.341) | (0.052) | (0.054) | (0.489) | (0.775) | (0.147) | (0.107) |
| *Occurred 2-3 years ago* | -0.250* | -0.059 | -0.009 | -0.068 | -0.723 | -1.061 | 0.038 | 0.049 |
|  | (0.112) | (0.213) | (0.049) | (0.049) | (0.439) | (0.906) | (0.129) | (0.090) |
| *Occurred 4-5 years ago* | -0.215* | 0.183 | -0.050 | -0.048 | 0.098 | -0.816 | 0.095 | 0.007 |
|  | (0.088) | (0.220) | (0.043) | (0.046) | (0.290) | (0.666) | (0.106) | (0.091) |

Table A9 *continued.*

| *Life event:* | Physically attacked | |
| --- | --- | --- |
|  | Poor | Rich |
| *Occurs in 4-5 years* | -0.054 | 0.176 |
|  | (0.205) | (0.282) |
| *Occurs in 2-3 years* | 0.140 | -0.129 |
|  | (0.207) | (0.234) |
| Occurred 0-1 years ago | -0.029 | 0.228 |
|  | (0.196) | (0.274) |
| *Occurred 2-3 years ago* | -0.056 | 0.478** |
|  | (0.152) | (0.185) |
| *Occurred 4-5 years ago* | 0.129 | 0.150 |
|  | (0.143) | (0.176) |

Table A10. Leads and Lags in Life Satisfaction to Negative Life Events by Hope Level, HILDA Survey 2007-2021

|  | Low hope | Full of hope |
| --- | --- | --- |
| ***Major worsening in finances*** |  |  |
| *Occurs in 4-5 years* | -0.617 | 0.002 |
|  | (0.410) | (0.092) |
| *Occurs in 2-3 years* | 0.031 | -0.092 |
|  | (0.613) | (0.101) |
| Occurred 0-1 years ago | -1.520** | -0.307** |
|  | (0.518) | (0.094) |
| *Occurred 2-3 years ago* | -0.805 | 0.054 |
|  | (0.543) | (0.065) |
| *Occurred 4-5 years ago* | -0.737 | 0.093 |
|  | (0.403) | (0.063) |
| No. of observations | 854 | 24,169 |
| No. of individuals | 628 | 9,558 |
| ***Fired or made redundant*** |  |  |
| *Occurs in 4-5 years* | -0.932 | -0.069 |
|  | (0.591) | (0.054) |
| *Occurs in 2-3 years* | -1.155* | -0.104 |
|  | (0.544) | (0.059) |
| Occurred 0-1 years ago | -0.748 | -0.140* |
|  | (0.683) | (0.062) |
| *Occurred 2-3 years ago* | -0.211 | -0.036 |
|  | (0.454) | (0.056) |
| *Occurred 4-5 years ago* | -0.106 | -0.023 |
|  | (0.668) | (0.057) |
| No. of observations | 849 | 24,140 |
| No. of individuals | 624 | 9,547 |
| ***Serious personal injury or illness*** |  |  |
| *Occurs in 4-5 years* | -0.148 | -0.012 |
|  | (0.370) | (0.029) |
| *Occurs in 2-3 years* | -0.068 | -0.018 |
|  | (0.424) | (0.033) |
| Occurred 0-1 years ago | -0.295 | -0.125** |
|  | (0.398) | (0.038) |
| *Occurred 2-3 years ago* | -0.654 | 0.036 |
|  | (0.350) | (0.035) |
| *Occurred 4-5 years ago* | 0.611 | 0.030 |
|  | (0.415) | (0.031) |
| No. of observations | 839 | 24,070 |
| No. of individuals | 620 | 9,533 |

*Notes:* Figures are coefficient estimates from fixed-effects models of overall life satisfaction by level of hope. ‘Low hope’ subsample includes respondents with a standardized level of hope ≤ 0.25 (on a 0-1 scale). ‘Full of hope’ subsample includes respondents with a standardized level of hope that equals 1. Standard errors clustered at the individual level are presented in parentheses. Overall life satisfaction measure ranges from 0 (totally dissatisfied) to 10 (totally satisfied) and is summarized in Table 2. Life event dummies are summarized in Table 3. Included in the life satisfaction equations but not shown are the covariates age, education, marital status, household composition, employment status, household income, long-term health status, neighbourhood SES index, homeownership status, residential area, and year dummies. * and ** denote statistical significance at the 5% and 1% levels, respectively.

Table A11. Estimated Effect of Locus of Control on Hope, HILDA Survey

|  | |  | Pooled OLS model | | Fixed-effects  model | |  |
| --- | --- | --- | --- | --- | --- | --- | --- |
| Standardized  Locus of Control | |  | 0.392 | ** | 0.279 | ** |  |
|  | |  | (0.004) |  | (0.006) |  |  |
| **Age group** | |  |  |  |  |  |  |
| 25-34 | |  | -0.003 |  | -0.006 |  |  |
|  | |  | (0.014) |  | (0.020) |  |  |
| 35-44 | |  | 0.055 | ** | 0.001 |  |  |
|  | |  | (0.016) |  | (0.028) |  |  |
| 45-54 | |  | 0.138 | ** | 0.021 |  |  |
|  | |  | (0.015) |  | (0.033) |  |  |
| 55-64 | |  | 0.225 | ** | 0.044 |  |  |
|  | |  | (0.016) |  | (0.039) |  |  |
| 65+ | |  | 0.373 | ** | 0.108 | * |  |
|  | |  | (0.019) |  | (0.046) |  |  |
| **Marital status** | |  |  |  |  |  |  |
| Married | |  | 0.098 | ** | 0.075 | ** |  |
|  | |  | (0.013) |  | (0.023) |  |  |
| De-facto | |  | 0.022 |  | 0.064 | ** |  |
|  | |  | (0.013) |  | (0.020) |  |  |
| Separated | |  | -0.042 |  | -0.056 |  |  |
|  | |  | (0.026) |  | (0.037) |  |  |
| Divorced | |  | -0.021 |  | -0.003 |  |  |
|  | |  | (0.020) |  | (0.036) |  |  |
| Widowed | |  | 0.084 | ** | -0.012 |  |  |
|  | |  | (0.022) |  | (0.042) |  |  |
| **Household composition** | |  |  |  |  |  |  |
| No. of children | |  | 0.010 | * | 0.006 |  |  |
|  | |  | (0.004) |  | (0.006) |  |  |
| No. of adults | |  | -0.000 |  | 0.004 |  |  |
|  | |  | (0.004) |  | (0.006) |  |  |
| **Education level** | |  |  |  |  |  |  |
| University degree | |  | 0.020 |  | -0.051 |  |  |
|  | |  | (0.010) |  | (0.032) |  |  |
| Diploma/certificate | |  | -0.005 |  | -0.072 | ** |  |
|  | |  | (0.010) |  | (0.026) |  |  |
| High school graduate | |  | 0.016 |  | -0.072 | ** |  |
|  | |  | (0.012) |  | (0.024) |  |  |
| **Employment status** | |  |  |  |  |  |  |
| Employed full-time | |  | 0.174 | ** | 0.063 | ** |  |
|  | |  | (0.012) |  | (0.015) |  |  |
| Employed part-time | |  | 0.127 | ** | 0.037 | * |  |
|  | |  | (0.012) |  | (0.014) |  |  |
| Unemployed | |  | -0.053 | * | -0.063 | * |  |
|  | |  | (0.023) |  | (0.028) |  |  |
| Retired | |  | 0.111 | ** | 0.030 |  |  |
|  | |  | (0.014) |  | (0.016) |  |  |
| **Household income** | |  |  |  |  |  |  |
| Log of HH income | |  | 0.022 | ** | 0.016 | * |  |
|  | |  | (0.006) |  | (0.008) |  |  |
| Neighborhood SES | |  | 0.007 | ** | 0.006 | * |  |
|  | |  | (0.001) |  | (0.003) |  |  |
| **Living status** | |  |  |  |  |  |  |
| Homeowner | |  | 0.065 | ** | 0.012 |  |  |
|  | |  | (0.009) |  | (0.012) |  |  |
| Lives in major city | |  | -0.011 |  | -0.036 |  |  |
|  | |  | (0.012) |  | (0.026) |  |  |
| Lives in inner regional | |  | -0.018 |  | -0.030 |  |  |
|  | |  | (0.012) |  | (0.025) |  |  |
| Intercept | |  | 3.988 | ** | 4.319 | ** |  |
|  | |  | (0.064) |  | (0.088) |  |  |
| Number of observations | |  | 56,548 |  | 56,548 |  |  |
| Number of individuals | |  |  |  | 22,889 |  |  |
|  |  | | | | | | |

*Notes:* Figures are estimated coefficients from cross-sectional pooled OLS and fixed-effects models of hope. Standard errors clustered at the individual level are presented in parentheses. The dependent hope variable ranges from 1 (totally hopeless) to 5 (totally hopeful). Locus of control questions are available in the HILDA data for years 2007, 2011, 2015, and 2019 only. The locus of control scale is summarized in Table 2. The included external-internal locus of control scale is standardized as a 0-1 variable, with those above the mean having higher than average internal locus of control. All included covariates are summarized in Tables A1 and A2. Included in all regressions but not shown are survey year dummies. * and ** denote statistical significance at the 5% and 1% levels, respectively.

Table A12. Leads and Lags in Hope to Negative Life Events by Locus of Control Type, HILDA Survey

| *Life event:* | Money lost | | Fired | | Separated | | Physically attacked | |
| --- | --- | --- | --- | --- | --- | --- | --- | --- |
|  | External LOC | Internal LOC | External LOC | Internal LOC | External LOC | Internal LOC | External LOC | Internal LOC |
| *Occurs in 2-3 years* | -0.150 | -0.001 | 0.120 | 0.033 | -0.182 | -0.009 | 0.127 | 0.153 |
|  | (0.101) | (0.087) | (0.115) | (0.040) | (0.114) | (0.044) | (0.167) | (0.123) |
| Occurred 0-1 years ago | -0.227** | -0.205* | -0.131 | 0.012 | -0.167 | -0.146* | -0.315* | 0.027 |
|  | (0.083) | (0.093) | (0.103) | (0.032) | (0.105) | (0.060) | (0.125) | (0.124) |
| *Occurred 2-3 years ago* | -0.152 | -0.049 | -0.000 | -0.014 | -0.175 | -0.038 | -0.040 | 0.205* |
|  | (0.093) | (0.071) | (0.101) | (0.028) | (0.110) | (0.050) | (0.148) | (0.083) |

*Notes:* Figures are estimated coefficients from two fixed-effects (within-person) models of hope for external and internal locus of control subsamples. External locus of control subsample captures individuals in the bottom 25% of the external-internal locus of control scale. Internal locus of control subsample captures individuals in the top 25% of the external-internal locus of control scale. The locus of control scale is summarized in Table 2. Locus of control questions are available in the HILDA data for years 2007, 2011, 2015, and 2019 only. Standard errors clustered at the individual level are presented in parentheses. The dependent hope variable ranges from 1 (totally hopeless) to 5 (totally hopeful). All reported life events are summarised in Table 3. Included in the hope equations but not shown are the covariates age, employment status, household income, and year dummies. Number of person-year observations equals 7,206 for external LOC subsample and 7,599 for internal LOC subsample. * and ** denote statistical significance at the 5% and 1% levels respectively.

Table A12 *continued.*

| *Life event:* | Death of spouse/child | | Illness | | Jailed | | Lost home | |
| --- | --- | --- | --- | --- | --- | --- | --- | --- |
|  | External LOC | Internal LOC | External LOC | Internal LOC | External LOC | Internal LOC | External LOC | Internal LOC |
| *Occurs in 2-3 years* | 0.040 | 0.039 | 0.005 | 0.018 | 0.129 | -0.011 | 0.049 | 0.020 |
|  | (0.142) | (0.041) | (0.056) | (0.023) | (0.374) | (0.032) | (0.113) | (0.045) |
| Occurred 0-1 years ago | -0.294 | -0.120 | -0.228** | -0.001 | -0.340 | -0.055 | -0.022 | -0.007 |
|  | (0.193) | (0.068) | (0.050) | (0.020) | (0.233) | (0.053) | (0.109) | (0.043) |
| *Occurred 2-3 years ago* | 0.032 | 0.028 | -0.079 | 0.009 | 0.078 | -0.019 | 0.152 | 0.055 |
|  | (0.186) | (0.026) | (0.058) | (0.020) | (0.429) | (0.027) | (0.108) | (0.032) |

Table A13. Estimated Effect of Current Hope on Future Outcomes – Poor Subsample, HILDA Survey

|  | Years into the future: | | |
| --- | --- | --- | --- |
|  | + 2 years | + 4 years | + 10 years |
| **Wellbeing:** |  |  |  |
| Life satisfaction | 0.822** | 0.776** | 0.689** |
|  | (0.062) | (0.075) | (0.121) |
|  | *N=*21,499 | *N=*17,315 | *N=*6,931 |
| Been a happy person | 0.682** | 0.608** | 0.397** |
|  | (0.044) | (0.053) | (0.087) |
|  | *N=*21,123 | *N=*17,017 | *N=*6,815 |
| **Education:** |  |  |  |
| University degree (bachelor’s) | 0.057** | 0.065** | 0.044 |
|  | (0.014) | (0.016) | (0.022) |
|  | *N=*12,629 | *N=*10,650 | *N=*4,949 |
| **Economic:** |  |  |  |
| Unemployed | -0.013 | -0.016 | -0.018 |
|  | (0.010) | (0.010) | (0.013) |
|  | *N=*12,629 | *N=*10,650 | *N=*4,949 |
| Earnings (disposable income) | 4435.0** | 5735.6** | 11561.1** |
|  | (1131.2) | (1401.2) | (2747.3) |
|  | *N=*12,816 | *N=*10,808 | *N=*5,035 |
| Neighbourhood SES | 0.063 | 0.101 | 0.495** |
|  | (0.050) | (0.070) | (0.132) |
|  | *N=*21,778 | *N=*17,539 | *N=*7,044 |
| Changed jobs | -0.026 | -0.012 | 0.007 |
|  | (0.013) | (0.013) | (0.019) |
|  | *N=*12,434 | *N=*10,493 | *N=*4,886 |
| Financial risk taking | 0.025 | -0.011 | -0.070 |
|  | (0.034) | (0.040) | (0.096) |
|  | *N=*9,852 | *N=*7,473 | *N=*1,474 |
| **Health:** |  |  |  |
| Poor health | -0.142** | -0.144** | -0.162** |
|  | (0.014) | (0.016) | (0.027) |
|  | *N=*20,983 | *N=*16,884 | *N=*6,731 |
| BMI: Obese | -0.028 | -0.032 | -0.053 |
|  | (0.012) | (0.015) | (0.026) |
|  | *N=*21,536 | *N=*17,339 | *N=*6,941 |
| Heavy drinker | -0.007 | 0.002 | 0.027 |
|  | (0.008) | (0.010) | (0.017) |
|  | *N=*21,536 | *N=*17,339 | *N=*6,941 |
| Heavy smoker | -0.045** | -0.040** | -0.031 |
|  | (0.009) | (0.011) | (0.019) |
|  | *N=*21,536 | *N=*17,339 | *N=*6,941 |
| Serious injury or illness | -0.070** | -0.063** | -0.050 |
|  | (0.012) | (0.014) | (0.021) |
|  | *N=*20,937 | *N=*16,884 | *N=*6,789 |
| **Social:** |  |  |  |
| Felt very lonely | -1.335** | -1.311** | -1.092** |
|  | (0.072) | (0.081) | (0.136) |
|  | *N=*20,971 | *N=*16,928 | *N=*6,761 |
| Have lots of friends | 0.468** | 0.502** | 0.420** |
|  | (0.056) | (0.066) | (0.111) |
|  | *N=*20,975 | *N=*16,932 | *N=*6,759 |
| Jailed | -0.008 | -0.004 | -0.015 |
|  | (0.003) | (0.003) | (0.006) |
|  | *N=*21,035 | *N=*16,960 | *N=*6,819 |

*Notes:* Prospective analysis of later life outcomes as a function of current feelings of hope for poor subsample. These regressions should be read horizontally. Poor subsample captures individuals in the bottom income quartile. For each listed outcome or dependent variable, the estimated coefficients are from three separate cross-sectional (pooled) OLS models predicting the outcome variable *t+2*, *t+4*, and *t+10* years into the future. Robust standard errors clustered at the individual level are presented in parentheses. The average age in the full sample is 45.4 years (ranging from 15 to 101). Age distribution of respondents: 17% (15-24); 17% (25-34); 16% (35-44); 17% (45-54); 15% (55-64); 11% (65-74); 7% (≥75 years old). For the economic and educational outcomes (except Neighborhood SES), we restrict the sample to individuals aged between 18 and 65. Included and controlled for in each prospective model but not shown is the level of the outcome variable in the current year *t* as well as the covariates age, education, marital status, household composition, employment status, household income, long-term health condition, neighborhood SES index, homeownership status, residential area, and year dummies. As an example, for the future outcome of graduating with a bachelor’s degree – the estimated model controls for and is conditional on the individual having graduated from high school in the current year. P-values are corrected using the Bonferroni method (see Bland and Altman 1995). In this table, we multiply the original p-values by 48; as we have 16 separate regression-equations (outcomes) over 3 time periods.

* and ** denote statistical significance at the 5% and 1% levels, respectively.

Table A14. Estimated Effect of Current Hope on Future Outcomes – Rich Subsample, HILDA Survey

|  | Years into the future: | | |
| --- | --- | --- | --- |
|  | + 2 years | + 4 years | + 10 years |
| **Wellbeing:** |  |  |  |
| Life satisfaction | 0.471** | 0.425** | 0.529** |
|  | (0.056) | (0.073) | (0.149) |
|  | *N=*20,017 | *N=*14,698 | *N=*4,488 |
| Been a happy person | 0.632** | 0.488** | 0.525** |
|  | (0.053) | (0.070) | (0.130) |
|  | *N=*19,846 | *N=*14,580 | *N=*4,462 |
| **Education:** |  |  |  |
| University degree (bachelor’s) | 0.035 | 0.027 | 0.101 |
|  | (0.025) | (0.030) | (0.050) |
|  | *N=*17,516 | *N=*12,961 | *N=*4,023 |
| **Economic:** |  |  |  |
| Unemployed | -0.013 | -0.005 | -0.038 |
|  | (0.008) | (0.009) | (0.018) |
|  | *N=*17,516 | *N=*12,961 | *N=*4,023 |
| Earnings (disposable income) | 15370.1** | 13337.9 | 40185.1** |
|  | (3507.0) | (5042.5) | (10682.7) |
|  | *N=*17,526 | *N=*12,970 | *N=*4,026 |
| Neighbourhood SES | 0.073 | 0.067 | 0.120 |
|  | (0.056) | (0.088) | (0.239) |
|  | *N=*20,015 | *N=*14,702 | *N=*4,488 |
| Changed jobs | -0.035 | -0.057 | -0.030 |
|  | (0.017) | (0.021) | (0.037) |
|  | *N=*17,413 | *N=*12,886 | *N=*4,005 |
| Financial risk taking | 0.051 | 0.106 | 0.113 |
|  | (0.032) | (0.044) | (0.111) |
|  | *N=*14,932 | *N=*10,478 | *N=*1,872 |
| **Health:** |  |  |  |
| Poor health | -0.127** | -0.119** | -0.191** |
|  | (0.015) | (0.019) | (0.040) |
|  | *N=*19,711 | *N=*14,468 | *N=*4,412 |
| BMI: Obese | -0.026 | -0.024 | -0.047 |
|  | (0.014) | (0.018) | (0.041) |
|  | *N=*20,022 | *N=*14,702 | *N=*4,491 |
| Heavy drinker | -0.001 | 0.005 | 0.052 |
|  | (0.011) | (0.014) | (0.029) |
|  | *N=*20,022 | *N=*14,702 | *N=*4,491 |
| Heavy smoker | -0.009 | -0.009 | -0.004 |
|  | (0.009) | (0.012) | (0.027) |
|  | *N=*20,022 | *N=*14,702 | *N=*4,491 |
| Serious injury or illness | -0.050** | -0.065** | -0.046 |
|  | (0.012) | (0.015) | (0.026) |
|  | *N=*19,896 | *N=*14,603 | *N=*4,465 |
| **Social:** |  |  |  |
| Felt very lonely | -1.259** | -1.163** | -1.134** |
|  | (0.081) | (0.101) | (0.192) |
|  | *N=*19,812 | *N=*14,533 | *N=*4,452 |
| Have lots of friends | 0.341** | 0.346** | 0.475 |
|  | (0.060) | (0.081) | (0.157) |
|  | *N=*19,830 | *N=*14,548 | *N=*4,454 |
| Jailed | -0.005 | -0.006 | -0.002 |
|  | (0.002) | (0.002) | (0.002) |
|  | *N=*19,910 | *N=*14,616 | *N=*4,470 |

*Notes:* Prospective analysis of later life outcomes as a function of current feelings of hope for rich subsample. These regressions should be read horizontally. Rich subsample captures individuals in the top income quartile. For each listed outcome or dependent variable, the estimated coefficients are from three separate cross-sectional (pooled) OLS models predicting the outcome variable *t+2*, *t+4*, and *t+10* years into the future. Robust standard errors clustered at the individual level are presented in parentheses. The average age in the full sample is 45.4 years (ranging from 15 to 101). Age distribution of respondents: 17% (15-24); 17% (25-34); 16% (35-44); 17% (45-54); 15% (55-64); 11% (65-74); 7% (≥75 years old). For the economic and educational outcomes (except Neighborhood SES), we restrict the sample to individuals aged between 18 and 65. Included and controlled for in each prospective model but not shown is the level of the outcome variable in the current year *t* as well as the covariates age, education, marital status, household composition, employment status, household income, long-term health condition, neighborhood SES index, homeownership status, residential area, and year dummies. As an example, for the future outcome of graduating with a bachelor’s degree – the estimated model controls for and is conditional on the individual having graduated from high school in the current year. P-values are corrected using the Bonferroni method (see Bland and Altman 1995). In this table, we multiply the original p-values by 48; as we have 16 separate regression-equations (outcomes) over 3 time periods.

* and ** denote statistical significance at the 5% and 1% levels, respectively.

| Table A15. Within-person estimates of participating in the next survey wave,  HILDA Survey 2007-2021 | | | | | | | | |
| --- | --- | --- | --- | --- | --- | --- | --- | --- |
|  | **Dependent variable:** Participated in the next survey wave (t+1) | | | | | | | |
| **Characteristics in survey wave t:** | (1) | | (2) | | (3) | | (4) | |
| Felt hopeful [1-5] | 0.005 | * | 0.009 | ** |  |  |  |  |
|  | (0.002) |  | (0.002) |  |  |  |  |  |
| High hope [0/1] |  |  |  |  | 0.009 |  | 0.015 | ** |
|  |  |  |  |  | (0.005) |  | (0.005) |  |
| Life Satisfaction | 0.010 | ** |  |  | 0.010 | ** |  |  |
|  | (0.001) |  |  |  | (0.001) |  |  |  |
| Age | -0.001 |  | -0.002 |  | -0.002 |  | -0.002 |  |
|  | (0.001) |  | (0.001) |  | (0.001) |  | (0.001) |  |
| Age-squared/100 | -0.012 | ** | -0.012 | ** | -0.012 | ** | -0.012 | ** |
|  | (0.001) |  | (0.001) |  | (0.001) |  | (0.001) |  |
| Married | -0.014 |  | -0.014 |  | -0.014 |  | -0.014 |  |
|  | (0.010) |  | (0.010) |  | (0.010) |  | (0.010) |  |
| De-facto | -0.002 |  | -0.001 |  | -0.002 |  | -0.001 |  |
|  | (0.008) |  | (0.008) |  | (0.008) |  | (0.008) |  |
| Separated | -0.015 |  | -0.016 |  | -0.015 |  | -0.016 |  |
|  | (0.015) |  | (0.015) |  | (0.015) |  | (0.015) |  |
| Divorced | 0.004 |  | 0.004 |  | 0.004 |  | 0.004 |  |
|  | (0.016) |  | (0.016) |  | (0.016) |  | (0.016) |  |
| Widowed | -0.028 |  | -0.029 |  | -0.028 |  | -0.029 |  |
|  | (0.019) |  | (0.019) |  | (0.019) |  | (0.019) |  |
| No. of children | 0.002 |  | 0.002 |  | 0.002 |  | 0.002 |  |
|  | (0.003) |  | (0.003) |  | (0.003) |  | (0.003) |  |
| No. of adults | 0.007 | ** | 0.007 | ** | 0.007 | ** | 0.007 | ** |
|  | (0.002) |  | (0.002) |  | (0.002) |  | (0.002) |  |
| University degree | -0.133 | ** | -0.135 | ** | -0.133 | ** | -0.135 | ** |
|  | (0.014) |  | (0.014) |  | (0.014) |  | (0.014) |  |
| Diploma | -0.109 | ** | -0.111 | ** | -0.109 | ** | -0.111 | ** |
|  | (0.011) |  | (0.011) |  | (0.011) |  | (0.011) |  |
| High school | -0.122 | ** | -0.123 | ** | -0.122 | ** | -0.123 | ** |
|  | (0.009) |  | (0.009) |  | (0.009) |  | (0.009) |  |
| Employed FT | -0.027 | ** | -0.027 | ** | -0.027 | ** | -0.027 | ** |
|  | (0.006) |  | (0.006) |  | (0.006) |  | (0.006) |  |
| Employed PT | -0.014 | ** | -0.013 | ** | -0.014 | ** | -0.013 | * |
|  | (0.005) |  | (0.005) |  | (0.005) |  | (0.005) |  |
| Unemployed | -0.009 |  | -0.010 |  | -0.009 |  | -0.010 |  |
|  | (0.009) |  | (0.009) |  | (0.009) |  | (0.009) |  |
| Retired | 0.001 |  | 0.003 |  | 0.002 |  | 0.003 |  |
|  | (0.006) |  | (0.006) |  | (0.006) |  | (0.006) |  |
| Log of HH income | -0.009 | ** | -0.008 | ** | -0.009 | ** | -0.008 | ** |
|  | (0.003) |  | (0.003) |  | (0.003) |  | (0.003) |  |
| Neighborhood SES | -0.000 |  | -0.000 |  | -0.000 |  | -0.000 |  |
|  | (0.001) |  | (0.001) |  | (0.001) |  | (0.001) |  |
| Homeowner | 0.008 |  | 0.009 |  | 0.008 |  | 0.009 |  |
|  | (0.005) |  | (0.005) |  | (0.005) |  | (0.005) |  |
| Major city | -0.007 |  | -0.008 |  | -0.007 |  | -0.008 |  |
|  | (0.011) |  | (0.011) |  | (0.011) |  | (0.011) |  |
| Inner regional | 0.002 |  | 0.002 |  | 0.002 |  | 0.002 |  |
|  | (0.011) |  | (0.011) |  | (0.011) |  | (0.011) |  |
| Long-term health condition | -0.002 |  | -0.004 |  | -0.003 |  | -0.004 |  |
|  | (0.004) |  | (0.004) |  | (0.004) |  | (0.004) |  |
| Smoke every day | -0.011 |  | -0.011 |  | -0.011 |  | -0.011 |  |
|  | (0.008) |  | (0.008) |  | (0.008) |  | (0.008) |  |
| Drink every day | 0.003 |  | 0.002 |  | 0.003 |  | 0.002 |  |
|  | (0.006) |  | (0.006) |  | (0.006) |  | (0.006) |  |
| BMI: Underweight | -0.023 |  | -0.023 |  | -0.023 |  | -0.023 |  |
|  | (0.013) |  | (0.013) |  | (0.013) |  | (0.013) |  |
| BMI: Healthy | -0.010 |  | -0.010 |  | -0.010 |  | -0.010 |  |
|  | (0.006) |  | (0.006) |  | (0.006) |  | (0.006) |  |
| BMI: Overweight | -0.002 |  | -0.002 |  | -0.002 |  | -0.002 |  |
|  | (0.005) |  | (0.005) |  | (0.005) |  | (0.005) |  |
| Money lost | -0.005 |  | -0.008 |  | -0.005 |  | -0.010 |  |
|  | (0.008) |  | (0.008) |  | (0.008) |  | (0.008) |  |
| Fired | -0.002 |  | -0.003 |  | -0.003 |  | -0.003 |  |
|  | (0.007) |  | (0.007) |  | (0.007) |  | (0.007) |  |
| Separated | -0.001 |  | -0.003 |  | -0.001 |  | -0.004 |  |
|  | (0.008) |  | (0.008) |  | (0.008) |  | (0.008) |  |
| Spouse or child died | -0.009 |  | -0.009 |  | -0.009 |  | -0.010 |  |
|  | (0.016) |  | (0.016) |  | (0.016) |  | (0.016) |  |
| Serious injury/illness | -0.002 |  | -0.004 |  | -0.002 |  | -0.004 |  |
|  | (0.005) |  | (0.005) |  | (0.005) |  | (0.005) |  |
| Physically attacked | 0.025 |  | 0.023 |  | 0.024 |  | 0.022 |  |
|  | (0.013) |  | (0.013) |  | (0.013) |  | (0.013) |  |
| Jailed | -0.027 |  | -0.028 |  | -0.027 |  | -0.028 |  |
|  | (0.035) |  | (0.035) |  | (0.035) |  | (0.035) |  |
| Lost home | -0.029 | ** | -0.028 | ** | -0.029 | ** | -0.029 | ** |
|  | (0.010) |  | (0.010) |  | (0.010) |  | (0.010) |  |
| Intercept | 1.290 | ** | 1.349 | ** | 1.304 | ** | 1.376 | ** |
|  | (0.046) |  | (0.045) |  | (0.046) |  | (0.044) |  |
| No. of observations | 87,358 |  | 87,390 |  | 87,358 |  | 87,390 |  |
| No. of individuals | 23,291 |  | 23,297 |  | 23,291 |  | 23,297 |  |
| *Notes*: The outcome variable is whether the respondent participates in the next wave of data collection (=1). ‘Felt hopeful’ variable is summarized in Table 2. ‘High hope’ equals 1 if survey respondents scored ≥4 (out of 5) on the ‘Felt hopeful’ scale, 0 otherwise. Demographic, health, employment, and income variables are described in Tables A1 and A2. Life event variables are summarized in Table 3. Standard errors clustered at the individual level are shown in parentheses.  * and ** denote statistical significance at the 5% and 1% levels, respectively. | | | | | | | | |

Table A16. Estimated Effect of Current Hope on Future Life Outcomes,

Balanced Panel, HILDA Survey 2007-2021

|  | *Years into the future:* | | |
| --- | --- | --- | --- |
| *Outcome variables:* | + 2 years | + 4 years | + 10 years |
| **Wellbeing:** |  |  |  |
| Life satisfaction | 0.628** | 0.681** | 0.712** |
|  | (0.048) | (0.058) | (0.082) |
|  | *N=37,450* | *N=32,105* | *N=16,036* |
| Been a happy person | 0.651** | 0.587** | 0.540** |
|  | (0.039) | (0.045) | (0.068) |
|  | *N=37,037* | *N=31,767* | *N=15,845* |
| **Education:** |  |  |  |
| University degree (bachelor’s) | 0.031 | 0.034 | 0.036 |
|  | (0.018) | (0.019) | (0.021) |
|  | *N=30,417* | *N=26,631* | *N=13,984* |
| **Economic:** |  |  |  |
| Unemployed | -0.013 | -0.017 | -0.016 |
|  | (0.006) | (0.007) | (0.009) |
|  | *N=30,417* | *N=26,631* | *N=13,984* |
| Earnings (disposable income) | 6878.1** | 9757.5** | 12869.3** |
|  | (1350.8) | (1925.0) | (3134.5) |
|  | *N=30,513* | *N=26,716* | *N=14,047* |
| Neighbourhood SES | 0.097 | 0.139 | 0.267 |
|  | (0.039) | (0.059) | (0.115) |
|  | *N=37,583* | *N=32,217* | *N=16,108* |
| Changed jobs | -0.027 | -0.020 | -0.018 |
|  | (0.011) | (0.012) | (0.015) |
|  | *N=30,210* | *N=26,459* | *N=13,887* |
| Financial risk taking | 0.049 | 0.053 | -0.032 |
|  | (0.024) | (0.030) | (0.065) |
|  | *N=21,433* | *N=17,092* | *N=4,219* |
| **Health:** |  |  |  |
| Poor health | -0.136** | -0.145** | -0.170** |
|  | (0.012) | (0.014) | (0.022) |
|  | *N=36,759* | *N=31,536* | *N=15,691* |
| BMI: Obese | -0.027 | -0.023 | -0.032 |
|  | (0.010) | (0.012) | (0.021) |
|  | *N=37,464* | *N=32,117* | *N=16,043* |
| Heavy drinker | 0.001 | -0.004 | -0.000 |
|  | (0.008) | (0.010) | (0.016) |
|  | *N=37,464* | *N=32,117* | *N=16,043* |
| Heavy smoker | -0.017 | -0.019 | -0.007 |
|  | (0.007) | (0.009) | (0.015) |
|  | *N=37,464* | *N=32,117* | *N=16,043* |
| Serious injury or illness | -0.063** | -0.055** | -0.051 |
|  | (0.011) | (0.011) | (0.015) |
|  | *N=37,068* | *N=31,795* | *N=15,881* |
| **Social:** |  |  |  |
| Felt very lonely | -1.307** | -1.318** | -1.202** |
|  | (0.061) | (0.069) | (0.103) |
|  | *N=36,984* | *N=31,736* | *N=15,822* |
| Have lots of friends | 0.374** | 0.418** | 0.382** |
|  | (0.043) | (0.052) | (0.082) |
|  | *N=37,015* | *N=31,766* | *N=15,844* |
| Jailed | -0.002 | -0.000 | -0.002 |
|  | (0.001) | (0.001) | (0.002) |
|  | *N=37,173* | *N=31,881* | *N=15,917* |

*Notes:* Prospective analysis of future life outcomes as a function of current feelings of hope. These regressions should be read horizontally. Analyzed sample is restricted to balanced panel of n=5,372 respondents who are present during the entire observation period (years 2007-2021); i.e., in each of the eight survey waves. For each listed dependent (outcome) variable, we estimate three separate pooled OLS models predicting the outcome variable *t+2*, *t+4*, and *t+10* years into the future. Robust standard errors clustered at the individual level are presented in parentheses. The average age in the full sample is 45.4 years (ranging from 15 to 101). Age distribution of respondents: 17% (15-24); 17% (25-34); 16% (35-44); 17% (45-54); 15% (55-64); 11% (65-74); 7% (≥75 years old). For the economic and educational outcomes (except Neighborhood SES), we restrict the sample to individuals aged between 18 and 65. Included and controlled for in each prospective model but not shown is the level of the outcome variable in the current year *t* as well as the covariates age, education, marital status, household composition, employment status, household income, long-term health condition, neighborhood SES index, homeownership status, residential area, and year dummies. As an example, for the future outcome of graduating with a bachelor’s degree – the estimated model controls for and is conditional on the individual having graduated from high school in the current year. P-values are corrected using the Bonferroni method (see Bland and Altman 1995). In this table, we multiply the original p-values by 48; as we have 16 separate regression-equations (outcomes) over 3 time periods.

* and ** denote statistical significance at the 5% and 1% levels, respectively.

Table A17. Estimated Effect of Current Hope on Future Life Outcomes –

with Lagged Self-Assessed Health as a Control, HILDA Survey 2007-2021

|  | *Years into the future:* | | |
| --- | --- | --- | --- |
| *Outcome variables:* | + 2 years | + 4 years | + 10 years |
| **Wellbeing:** |  |  |  |
| Life satisfaction | 0.558** | 0.530** | 0.603** |
|  | (0.030) | (0.038) | (0.065) |
|  | *N=84,141* | *N=66,369* | *N=25,239* |
| Been a happy person | 0.600** | 0.502** | 0.478** |
|  | (0.024) | (0.029) | (0.051) |
|  | *N=83,177* | *N=65,630* | *N=24,958* |
| **Education:** |  |  |  |
| University degree (bachelor’s) | 0.036* | 0.038* | 0.043 |
|  | (0.011) | (0.012) | (0.016) |
|  | *N=66,387* | *N=53,225* | *N=21,360* |
| **Economic:** |  |  |  |
| Unemployed | -0.022** | -0.018** | -0.016 |
|  | (0.005) | (0.005) | (0.007) |
|  | *N=66,387* | *N=53,225* | *N=21,360* |
| Earnings (disposable income) | 5714.2** | 6175.7** | 15387.8** |
|  | (946.8) | (1292.7) | (2485.7) |
|  | *N=66,591* | *N=53,397* | *N=21,450* |
| Neighbourhood SES | 0.046 | 0.098 | 0.143 |
|  | (0.027) | (0.040) | (0.088) |
|  | *N=84,420* | *N=66,594* | *N=25,357* |
| Changed jobs | -0.032** | -0.030** | -0.011 |
|  | (0.008) | (0.008) | (0.013) |
|  | *N=65,789* | *N=52,770* | *N=21,183* |
| Financial risk taking | 0.015 | 0.034 | -0.044 |
|  | (0.017) | (0.021) | (0.054) |
|  | *N=49,395* | *N=36,819* | *N=7,359* |
| **Health:** |  |  |  |
| Poor health | -0.138** | -0.142** | -0.158** |
|  | (0.007) | (0.009) | (0.016) |
|  | *N=83,388* | *N=65,820* | *N=25,025* |
| BMI: Obese | -0.017 | -0.007 | -0.021 |
|  | (0.007) | (0.008) | (0.016) |
|  | *N=84,197* | *N=66,409* | *N=25,261* |
| Heavy drinker | 0.0004 | 0.008 | 0.011 |
|  | (0.005) | (0.006) | (0.012) |
|  | *N=84,197* | *N=66,409* | *N=25,261* |
| Heavy smoker | -0.030** | -0.035** | -0.022 |
|  | (0.005) | (0.006) | (0.012) |
|  | *N=84,197* | *N=66,409* | *N=25,261* |
| Serious injury or illness | -0.036** | -0.043** | -0.039* |
|  | (0.006) | (0.007) | (0.012) |
|  | *N=82,979* | *N=65,518* | *N=24,953* |
| **Social:** |  |  |  |
| Felt very lonely | -1.200** | -1.157** | -1.060** |
|  | (0.039) | (0.046) | (0.080) |
|  | *N=82,866* | *N=65,400* | *N=24,877* |
| Have lots of friends | 0.346** | 0.348** | 0.339** |
|  | (0.029) | (0.036) | (0.065) |
|  | *N=82,927* | *N=65,457* | *N=24,892* |
| Jailed | -0.004* | -0.003 | -0.006 |
|  | (0.001) | (0.001) | (0.003) |
|  | *N=83,209* | *N=65,686* | *N=25,012* |

*Notes:* Prospective analysis of future life outcomes as a function of current feelings of hope. These regressions should be read horizontally. For each listed dependent (outcome) variable, we estimate three separate pooled OLS models predicting the outcome variable *t+2*, *t+4*, and *t+10* years into the future. Robust standard errors clustered at the individual level are presented in parentheses. The average age in the full sample is 45.4 years (ranging from 15 to 101). Age distribution of respondents: 17% (15-24); 17% (25-34); 16% (35-44); 17% (45-54); 15% (55-64); 11% (65-74); 7% (≥75 years old). For the economic and educational outcomes (except Neighborhood SES), we restrict the sample to individuals aged between 18 and 65. Included and controlled for in each prospective model but not shown is the level of the outcome variable in the current year *t* as well as the covariates age, education, marital status, household composition, employment status, household income, self-assessed health, long-term health condition, neighborhood SES index, homeownership status, residential area, and year dummies. As an example, for the future outcome of graduating with a bachelor’s degree – the estimated model controls for and is conditional on the individual having graduated from high school in the current year. P-values are corrected using the Bonferroni method (see Bland and Altman 1995). In this table, we multiply the original p-values by 48; as we have 16 separate regression-equations (outcomes) over 3 time periods.

* and ** denote statistical significance at the 5% and 1% levels, respectively.
